# Flexible Signal Denoising via Flexible Empirical Bayes Shrinkage

**Published:** 2021

**Authors:** Zhengrong Xing, Peter Carbonetto, Matthew Stephens

**Affiliations:** Department of Statistics, University of Chicago, Chicago, IL 60637, USA; Research Computing Center and Department of Human Genetics, University of Chicago, Chicago, IL 60637, USA; Department of Statistics and Department of Human Genetics, University of Chicago, Chicago, IL 60637, USA

**Keywords:** Empirical Bayes, wavelets, non-parametric regression, mean estimation, variance estimation

## Abstract

Signal denoising—also known as non-parametric regression—is often performed through shrinkage estimation in a transformed (e.g., wavelet) domain; shrinkage in the transformed domain corresponds to smoothing in the original domain. A key question in such applications is how much to shrink, or, equivalently, how much to smooth. Empirical Bayes shrinkage methods provide an attractive solution to this problem; they use the data to estimate a distribution of underlying “effects,” hence automatically select an appropriate amount of shrinkage. However, most existing implementations of empirical Bayes shrinkage are less flexible than they could be—both in their assumptions on the underlying distribution of effects, and in their ability to handle heteroskedasticity—which limits their signal denoising applications. Here we address this by adopting a particularly flexible, stable and computationally convenient empirical Bayes shrinkage method and applying it to several signal denoising problems. These applications include smoothing of Poisson data and heteroskedastic Gaussian data. We show through empirical comparisons that the results are competitive with other methods, including both simple thresholding rules and purpose-built empirical Bayes procedures. Our methods are implemented in the R package smashr, “SMoothing by Adaptive SHrinkage in R,” available at https://www.github.com/stephenslab/smashr.

## 1. Introduction

Shrinkage and sparsity play key roles in many areas of modern statistics, including high-dimensional regression (Tibshirani, 1996), covariance or precision matrix estimation (Bickel and Levina, 2008), multiple testing (Efron, 2004) and signal denoising (Donoho and Johnstone, 1994, 1995). One attractive way to achieve shrinkage and sparsity is via Bayesian or empirical Bayes (EB) methods (e.g., Efron and Tibshirani, 2002; Johnstone and Silverman, 2004, 2005b; Clyde and George, 2000; Daniels and Kass, 2001). These methods are attractive because they can adapt the amount of shrinkage to the data. Specifically, by learning the distribution of the underlying “effects” that are being estimated, EB methods can appropriately adapt the amount of shrinkage from data set to data set, and indeed from data point to data point. For example, in settings where the effects are sparse, but with a long tail of large effects, optimal accuracy is achieved by strongly shrinking observations that lie near zero while minimally shrinking the strongest signals (Polson and Scott, 2010). This form of shrinkage can be achieved by suitable EB methods.

One area where Bayesian methods for shrinkage have been found to be particularly effective is in signal denoising (Abramovich et al., 1998; Clyde and George, 2000; Johnstone and Silverman, 2005b). Shrinkage plays a key role in signal denoising because signal denoising can be accurately and conveniently achieved by shrinkage in a transformed (e.g., wavelet) domain (Donoho and Johnstone, 1994). In empirical comparisons (e.g., Antoniadis et al., 2001; Besbeas et al., 2004), Bayesian methods often outperform alternatives such as simple thresholding rules (Coifman and Donoho, 1995; Donoho and Johnstone, 1994). However, existing software implementations of Bayesian and EB methods for this problem are limited; for example, the ebayesthresh. wavelet function in the R package EbayesThresh (Johnstone and Silverman, 2005a) only implements methods for the particular case of estimating Gaussian means with constant variance.

Here we show how EB shrinkage can easily be applied to other signal denoising problems. The key to this generalization is, in essence, to use a more flexible EB shrinkage method that—among other benefits—allows for heteroskedastic variances (Stephens, 2017). This in turn allows it to tackle signal-denoising problems with heteroskedastic variances. We provide methods and software implementations for denoising Gaussian means in the presence of heteroskedastic variances, denoising Gaussian variances, and denoising Poisson means. These are all settings that are relatively underserved by existing implementations. Indeed, we are unaware of any existing EB implementation for wavelet denoising of either the mean or the variance in the heteroskedastic Gaussian case. Consistent with previous studies (Antoniadis et al., 2001; Besbeas et al., 2004), we find that the EB methods are more accurate than commonly used thresholding rules, and, in the Poisson case, competitive with a dedicated EB method (Kolaczyk, 1999). Our methods are implemented in the R package smashr (“SMoothing by Adaptive SHrinkage in R”), available on GitHub (https://www.github.com/stephenslab/smashr).

## 2. Background

Here we briefly review EB shrinkage methods, and show how they can be applied to a simple signal denoising application—Gaussian data with constant variance. The mathematical development mirrors Johnstone and Silverman (2005b).

### 2.1. Empirical Bayes Shrinkage

Consider observations $x=\left(x_{1},\ldots ,x_{p}\right)$ of underlying quantities $\theta =\left(\theta_{1},\ldots ,\theta_{p}\right)$, with Gaussian errors having standard deviation $s=\left(s_{1},\ldots ,s_{p}\right)$ for which we assume, for now, are known; that is,

(1) $$x|\theta \sim N_{p}\left(\theta ,\Delta \right)$$

where Δ is the diagonal matrix with diagonal entries $s_{1}^{2},\ldots ,s_{p}^{2}$. Although it is conceptually straightforward to allow the standard deviations $s_{j}$ to vary, in practice most treatments (and software implementations) assume them to be constant, $s_{j}=s$, an issue we return to later. The goal is to estimate θ. This is sometimes called the “normal means” problem.

Without any assumptions on θ, the natural estimate for θ seems to be the maximum likelihood estimate x. However, James and Stein (1961) showed that more accurate estimates can be obtained by using “shrinkage”, which essentially reduces variance at the cost of introducing some bias.

An attractive way to perform shrinkage in practice is to use EB methods. These methods assume that θ are independent and identically distributed from some (unknown) underlying distribution, g, which is further assumed to belong to some specified family of distributions 𝒢. Combining this with (1) yields:

(2) $$x|\theta \sim N_{p}\left(\theta ,\Delta \right),$$

(3) $$\theta_{1},\ldots ,\theta_{p}\overset{i.i.d.}{\sim }g\left(\cdot \right),\;g\in 𝒢.$$

EB methods estimate θ in two steps:

1. Estimate g by maximum likelihood,
$$\hat{g}=\underset{g\in 𝒢}{\text{argmax }}L\left(g\right),$$
  where
(4) $$L\left(g\right):=p\left(x|g\right)=\overset{p}{\underset{j=1}{\prod }}\int p\left(x_{j}|\theta_{j},s_{j}\right)g\left(d\theta_{j}\right).$$
2. Estimate each $\theta_{j}$ using its posterior distribution given $\overset{ˆ}{g}$,
(5) $$p\left(\theta_{j}|x,s,\hat{g}\right)\propto p\left(x_{j}|\theta_{j},s_{j}\right)\hat{g}\left(\theta_{j}\right).$$

We estimate $\theta_{j}$ using the mean of this posterior distribution. (One can also use the posterior median, which, if $\overset{ˆ}{g}$ has a point mass at zero, has a “thresholding” property; see Johnstone and Silverman 2005b. However, we have not found this necessary to achieve good performance in practice.) A key feature of EB methods is that, by estimating g from the data, they can adapt to each individual data set, essentially learning how much to shrink from the available data.

Different EB approaches differ in their assumptions on the family 𝒢, and which assumptions are most appropriate may depend on the setting. In many settings, including those of interest here, it is anticipated that θ may be “sparse”, with many entries at or near zero. This can be captured by restricting 𝒢 to “sparsityinducing” distributions that are unimodal at zero. For example, the Ebayes Thresh package (Johnstone and Silverman, 2005a) implements two options: (1) g is a mixture of a point mass at zero and a Laplace (or double exponential) distribution; or (2) g is a mixture of a point mass at zero and a Cauchy distribution. Another common assumption is that g is a mixture of a point mass at zero and a zero-mean Gaussian distribution, sometimes referred to as a “spike and slab” prior (Clyde and George, 2000).

Here we use the flexible “adaptive shrinkage” (ASH) EB methods introduced in Stephens (2017). These methods allow for more flexible distributional families 𝒢 while maintaining sparsity-inducing behaviour, and allow the standard deviations $s_{j}$ to vary. They are also computationally stable and efficient. When most flexible, ASH assumes 𝒢 to be the family of all unimodal distributions (with their modes set to zero in settings where sparsity is desired). Here we adopt a slightly more restrictive family, in which 𝒢 is the family of zerocentered scale mixtures of normals. In practice, this is achieved by using finite mixtures with a potentially large number of components; that is,

(6) $$g\left(\cdot \right)=\overset{K}{\underset{k=0}{\sum }}\pi_{k}N\left(\cdot ;0,\omega_{k}^{2}\right),$$

where the mixture weights $\pi_{0},\ldots ,\pi_{K}$ are non-negative and sum to 1, and $N\left(\cdot ;\mu ,\sigma^{2}\right)$ denotes the density of the normal distribution with mean μ and variance $\sigma^{2}$.

A key idea, which substantially simplifies inference, is to take $\omega_{0},\ldots ,\omega_{K}$ to be a fixed grid of values ranging from very small (e.g., $\omega_{0}=0$, in which case g includes a point mass at zero) to very large. Maximizing the likelihood (4) then becomes a convex optimization problem in π, which can be solved efficiently using interior point methods (Koenker and Mizera, 2014), sequential quadratic programming methods (Kim et al., 2020), or, more simply—though less efficiently for large problems—using accelerated EM algorithms (Henderson and Varadhan, 2019; Varadhan and Roland, 2008). The conditional distributions $p\left(\theta_{j}|x,s,\overset{ˆ}{g}\right)$ are analytically tractable, and the posterior mean $E\left(\theta_{j}|x,s,\overset{ˆ}{g}\right)$ provides a shrinkage point estimate for $\theta_{j}$. See Stephens (2017) for details and various embellishments, including generalizing the normal likelihood to a t likelihood.

The representation (6) provides a flexible family of unimodal and symmetric distributions. Indeed, with a sufficiently large and dense grid $\omega_{0},\ldots ,\omega_{K}$, the distribution g in (6) can approximate a scale mixture of normals to at any level of accuracy. This family includes, as a special case, the distributions used in Clyde and George (2000), Johnstone and Silverman (2005b), and many others (e.g., the Horseshoe prior of Carvalho et al., 2010). In this sense, ASH is more flexible than these existing EB approaches. Further, in many ways this approach *simplifies* inference; by fixing the $\omega_{k}$ on a dense grid, maximizing the likelihood (4) becomes a convex optimization problem.

It is possible to implement EB methods for even broader families, 𝒢. Indeed, Koenker and Mizera (2014), Koenker and Gu (2017) provide methods and software for a fully non-parametric solution; that is, 𝒢 is the set of all distributions on the real line. However, the resulting maximum likelihood estimate $\overset{ˆ}{g}$ is then discrete, which in the setting we consider here is unrealistic. More generally, in many settings—including those considered here—shrinkage towards zero is a desired outcome, and restricting 𝒢 to distributions that are unimodal at zero seems an attractive and flexible way to achieve this.

### 2.2. Signal Denoising via EB Shrinkage

Here we introduce the homoskedastic Gaussian non-parametric regression problem and summarize how it can be solved using the EB shrinkage methods as in Johnstone and Silverman (2005b).

The homoskedastic Gaussian non-parametric regression problem has essentially the same structure as the homoskedastic normal means problem (1), with the crucial difference that the means to be estimated, denoted $\mu ={\left(\mu_{1},\ldots ,\mu_{T}\right)}^{⊤}$, are expected to spatially structured. By spatially structured, we mean that $\mu_{t}$ will often be similar to $\mu_{t^{*}}$ for small $\left|t-t^{*}\right|$, although we do not rule out occasional abrupt changes in μ. In other words, homoskedastic Gaussian non-parametric regression involves estimating a spatially structured mean $\mu ={\left(\mu_{1},\ldots ,\mu_{T}\right)}^{⊤}$ from Gaussian observations $y={\left(y_{1},\ldots ,y_{T}\right)}^{⊤}$ with standard error σ,

(7) $$y|\mu \sim N_{T}\left(\mu ,\sigma^{2}I_{T}\right),$$

where $I_{T}$ is the T×T identity matrix. Here, t=1,…,T indexes location in a one-dimensional space, such as time or, as in a later example, position along the genome. For convenience, we assume $T=2^{J}$ for some integer J, which is a common assumption in multi-scale analyses.

Although the assumption that μ is spatially structured is very different from the sparsity assumption made by the EB shrinkage methods described above, EB shrinkage methods can nonetheless be used to solve this non-parametric regression problem (Johnstone and Silverman, 2005b). The key idea is to apply a discrete wavelet transform (DWT) to (7). The DWT can be expressed using an orthogonal T×T matrix W that depends on the wavelet basis chosen. Pre-multiplying (7) by W yields

(8) $$Wy|W\mu \sim N_{T}\left(W\mu ,\sigma^{2}WW^{⊤}\right).$$

Note that $WW^{⊤}=I_{T}$, so we write this as

(9) $$\overset{˜}{y}|\overset{˜}{\mu }\sim N_{T}\left(\overset{˜}{\mu },\sigma^{2}I_{T}\right),$$

in which $\tilde{y}:=Wy={\left({\tilde{y}}_{1},\ldots ,{\tilde{y}}_{T}\right)}^{⊤}$ are the empirical wavelet coefficients (WCs), and $\tilde{\mu }:=W\mu =$ ${\left({\tilde{\mu }}_{1},\ldots ,{\tilde{\mu }}_{T}\right)}^{⊤}$ are the (unknown) wavelet coefficients to be estimated.

A key feature of the DWT is that if μ is spatially structured, many of the wavelet coefficients $\tilde{\mu }$ will be close to zero, and vice versa (Mallat, 2009). Thus, the DWT has changed the problem from fitting (7) under the assumption that μ spatially structured to fitting (9) under the assumption that many of the WCs $\tilde{\mu }$ will be close to zero (Donoho and Johnstone, 1995). This is easily achieved by the sparsity-inducing EB shrinkage methods described above; it simply requires setting $x\leftarrow \tilde{y},\theta \leftarrow \tilde{\mu },s_{j}^{2}\leftarrow \sigma^{2}$, for j=1,…,T, and choosing 𝒢 to capture the assumption that g has most of its mass near zero. The value of σ is of course typically unknown, but it can be estimated by a number of simple methods (e.g., equation 2 or 3 from Brown and Levine, 2007). In practice, it is important to group the WCs by their resolution level before shrinking; see the note below.

The EB procedure yields shrinkage estimates, $\overset{ˆ}{\tilde{\mu }}$, of the WCs $\tilde{\mu }$, which can be reverse-transformed to obtain estimates of μ

(10) $$\overset{ˆ}{\mu }:=W^{-1}\overset{ˆ}{\overset{˜}{\mu }}=W^{T}\overset{ˆ}{\overset{˜}{\mu }}\text{.}$$

This outlines the basic strategy used by Johnstone and Silverman (2005b) implemented in the R package EbayesThresh (Johnstone and Silverman, 2005a).

## 3. Methods

Here, we extend the ideas from Johnstone and Silverman (2005b) for the homoskedastic Gaussian case and apply them to more general signal denoising settings. First, we consider Gaussian data with spatially structured mean *and* spatially structured variance (Section 3.1). In this setting, our methods provide estimates for both the mean and variance. Second, we consider denoising Poisson data (Section 3.2). In this setting, the variance depends on the mean, so a spatially structured mean implies spatially structured variance. Both settings require shrinkage methods that can deal with heteroskedastic errors, so we use the ASH method from Stephens (2017). We call these methods SMASH, an abbreviation of “SMoothing by Adaptive SHrinkage.”

### 3.1. Heteroskedastic Gaussian Data

The heteroskedastic analog of (7) is

(11) $$y|\mu \sim N_{T}\left(\mu ,D\right),$$

where D is the diagonal matrix with diagonal entries $\sigma^{2}=\left(\sigma_{1}^{2},\ldots ,\sigma_{T}^{2}\right)$.

Our goal here is to fit (11) when both μ and $\sigma^{2}$ are spatially structured. We consider, in turn, (i) estimating μ when $\sigma^{2}$ is known, (ii) estimating $\sigma^{2}$ when μ is known, and (iii) estimating μ and $\sigma^{2}$ when both are unknown.

#### 3.1.1. EStimating μ With $\sigma^{2}$ KNOWN

As in the homoskedastic case, the first step is to transform (11) using a wavelet transform,

(12) $$Wy|W\mu \sim N_{T}\left(W\mu ,WDW^{⊤}\right),$$

which we write as

(13) $$\overset{˜}{y}|\overset{˜}{\mu }\sim N_{T}\left(\overset{˜}{\mu },WDW^{⊤}\right).$$

As before, the $\tilde{y}:=Wy={\left({\tilde{y}}_{1},\ldots ,{\tilde{y}}_{T}\right)}^{⊤}$ are the empirical WCs, and the $\tilde{\mu }:=W\mu ={\left({\tilde{\mu }}_{1},\ldots ,{\tilde{\mu }}_{T}\right)}^{⊤}$ are the unknown WCs to be estimated. Unlike the homoskedastic case, the covariance matrix of the empirical WCs in (13) is no longer diagonal and, in particular, the diagonal entries (i.e., the variances) are no longer the same.

To account for different variances among the WCs, we apply EB shrinkage to the marginal distributions from (13),

(14) $${\tilde{y}}_{j}|{\tilde{\mu }}_{j}\sim N\left({\tilde{\mu }}_{j},\omega_{j}^{2}\right),$$

in which

(15) $$\omega_{j}^{2}=\overset{T}{\underset{t=1}{\sum }}\sigma_{t}^{2}W_{jt}^{2},\;j=1,\ldots ,T.$$

Specifically, to obtain estimates ${\overset{ˆ}{\tilde{\mu }}}_{j}$, we apply ASH (Section 2.1), which fits a large mixture of unimodal distributions, g, to the data, $x_{j}\leftarrow {\tilde{y}}_{j},s_{j}^{2}\leftarrow \omega_{j}^{2}$, for j=1,…,T. As in the homoskedastic case (Section 2.2), applying EB shrinkage to the WCs yields posterior mean estimates ${\overset{ˆ}{\tilde{\mu }}}_{j}$, from which estimates ${\overset{ˆ}{\mu }}_{j}$ are obtained by inverting the wavelet transform (10). Although this strategy accounts for heteroskedacity in the WCs, it ignores correlations among them. We are not alone in making this simplification; see Silverman (1999) for example.

The simple but crucial point here is that the shrinkage step requires EB methods that can solve the normal means problem with heteroskedastic variances. Most treatments of the normal means problem, including EbayesThresh, avoid this complication, whereas ASH is well suited to handling this situation.

#### 3.1.2. ESTIMATING $\sigma^{2}$ WITH μ KNOWN

To estimate the variances $\sigma^{2}=\left(\sigma_{1}^{2},\ldots ,\sigma_{T}^{2}\right)$ we apply wavelet shrinkage methods to the squared deviations from the mean, similar to the approaches of Delouille et al. (2004) and Cai and Wang (2008). Specifically, we define

(16) $$Z_{t}^{2}:={\left(y_{t}-\mu_{t}\right)}^{2},$$

and note that $E\left(Z_{t}^{2}\right)=\sigma_{t}^{2}$, so that estimating $\sigma^{2}$ reduces to a mean estimation problem with “observations” $Z^{2}:=\left(Z_{1}^{2},\ldots ,Z_{T}^{2}\right)$.

As in the procedure for estimating μ given $\sigma^{2}$ (Section 3.1.1), we estimate $\sigma^{2}$ by fitting the ASH model (Section 2.1) to the observations $x_{t}\leftarrow Z_{t}^{2}$. To apply ASH, we need an estimate of the variance of each $Z_{t}^{2}$. We use $s_{t}^{2}=\frac{2}{3}Z_{t}^{4}$, which is an unbiased estimator of the variance. (If $Z^{2}\sim \sigma^{2}\chi_{1}^{2}$, then $E\left(Z^{4}\right)=3\sigma^{4}$ and $\left.Var\;\left(Z^{2}\right)=2\sigma^{4}.\right)$

This approach effectively approximates the wavelet-transformed values ${\tilde{Z}}^{2}:=WZ^{2}={\left({\tilde{Z}}_{1}^{2},\ldots ,{\tilde{Z}}_{T}^{2}\right)}^{⊤}$ by a Gaussian distribution when really they are linear combinations of $\chi_{1}^{2}$ random variables. Despite this approximation, we have found this procedure to work well in practice in most cases, perhaps with a tendency to oversmooth quickly varying variance functions.

#### 3.1.3. ESTIMATING μ AND $\sigma^{2}$ JOINTLY

To deal with the (more common) case in which both mean and variance are unknown, we simply iterate the above procedures. That is, the algorithm consists of repeating the following two steps:

1. Estimate μ as if $\sigma^{2}$ is known (with $\sigma^{2}$ set to the estimate ${\overset{ˆ}{\sigma }}^{2}$ obtained from the previous iteration).
2. Estimate $\sigma^{2}$ as if μ is known (with μ set to the estimate ${\overset{ˆ}{\mu }}^{2}$ obtained from Step 1).

To initialize the algorithm, we estimate the variance $\sigma^{2}$ as

$${\hat{\sigma }}_{t}^{2}=\frac{1}{2}\left({\left(y_{t}-y_{t-1}\right)}^{2}+{\left(y_{t}-y_{t+1}\right)}^{2}\right),\;t=1,\ldots ,T,$$

defining $y_{0}=y_{n}$ and $y_{T+1}=y_{1}$ (equivalent to placing the locations on a circle).

We cannot guarantee that this procedure will converge, but in our simulations we found that two iterations of steps 1 and 2 reliably produced accurate results. (So the full procedure consists of initialization, running steps 1 and 2, then running steps 1 and 2 a second time.)

### 3.2. Poisson Data

Now we consider estimating a spatially structured mean $\mu ={\left(\mu_{1},\ldots ,\mu_{T}\right)}^{⊤}$ from Poisson data,

$$y_{t}\sim \text{Pois}\left(\mu_{t}\right),\;t=1,\ldots ,T.$$

For Poisson data, the analogue of the DWT is provided by the Poisson multiscale models from Kolaczyk (1999); Timmermann and Nowak (1999); Nowak and Kolaczyk (2000). In brief, we estimate μ by applying ASH to shrink the parameters within these multi-scale models.

To motivate this approach, first recall the following elementary distributional result: if $y_{1}$ and $y_{2}$ are independent, with $y_{t}\sim \text{Pois}\;\left(\mu_{t}\right)$ then

$$\begin{array}{rr} y_{1}+y_{2} & \sim \text{Pois}\left(\mu_{1}+\mu_{2}\right)\\ y_{1}|\left(y_{1}+y_{2}\right) & \sim \text{Bin}\left(y_{1}+y_{2},\mu_{1}/\left(\mu_{1}+\mu_{2}\right)\right). \end{array}$$

To extend this to T=2×2=4, we introduce notation $v_{i:j}$ to denote the sum $v_{i:j}=\sum_{t=i}^{j}\;v_{t}$ for some vector v. Then we have that

(17) $$y_{1:4}\sim \text{Pois}\left(\mu_{1:4}\right)$$

(18) $$y_{1:2}|y_{1:4}\sim \text{Bin}\left(y_{1:4},\mu_{1:2}/\mu_{1:4}\right)$$

(19) $$y_{1}|y_{1:2}\sim \text{Bin}\left(y_{1:2},\mu_{1}/\mu_{1:2}\right)$$

(20) $$y_{3}|y_{3:4}\sim \text{Bin}\left(y_{3:4},\mu_{3}/\mu_{3:4}\right).$$

Together, these models are equivalent to $y_{t}\sim \text{Pois}\;\left(\mu_{t}\right)$, for t=1,…,4, and they decompose the overall distribution $y_{1},\ldots ,y_{4}$ into parts involving aspects of the data at increasing resolution; (17) represents the coarsest resolution (the sum of all the data points), whereas (19, 20) represent the finest resolution, and (18) is the in-between resolution. This representation suggests a reparameterization, from $\left(\mu_{1},\mu_{2},\mu_{3},\mu_{4}\right)$ to $\left(\mu_{1:4},p\right)$, where binomial parameters $p=\left(p_{1},p_{2},p_{3}\right)=\left(\mu_{1:2}/\mu_{1:4},\mu_{1}/\mu_{1:2},\mu_{3}/\mu_{3:4}\right)$ control lower $\left(p_{1}\right)$ and higher resolution $\left(p_{2},p_{3}\right)$ changes in the mean vector μ. This idea extends naturally to $T=2^{J}$ for any J, reparameterizing μ into its sum $\mu_{1:T}$ and the T-1 binomial probabilities $p=\left(p_{1},\ldots ,p_{T-1}\right)$ that capture features of μ at different resolutions. This can be viewed as the Poisson analogue of the Haar wavelet transform.

In this reparameterization, $p_{j}=\frac{1}{2}$, for j=1,…,T-1, corresponds to the case of a constant mean vector, and values of $p_{j}$ far from $\frac{1}{2}$ correspond to large changes in μ at some scales. Therefore, estimating a spatially structured μ can be achieved by shrinkage estimation of p, with shrinkage towards $p_{j}=\frac{1}{2}$. Both Kolaczyk (1999) and Timmermann and Nowak (1999) use dedicated Bayesian models to achieve this shrinkage by introducing a prior distribution on elements of p that is a mixture of a point mass at $\frac{1}{2}$ (resulting in shrinkage toward $\frac{1}{2}$) and a Beta distribution. We take a different approach, reparameterizing the $p_{j}$ ‘s as $\alpha_{j}=log\;\left(\frac{p_{j}}{1-p_{j}}\right),j=1,\ldots ,T-1$, then using ASH to shrink the parameters $\alpha_{j}$ towards zero, since $\alpha_{j}=0$ when $p_{j}=\frac{1}{2}$. Since ASH is based on solving the normal means problem, this is effectively making a normal approximation to the likelihood for the parameters $\alpha_{j}$ (which is not the same as making a normal approximation for the data).

To obtain a normal approximation to the likelihood for $\alpha =\left(\alpha_{1},\ldots ,\alpha_{T-1}\right)$, it suffices to have an estimate ${\overset{ˆ}{\alpha }}_{j}$ and corresponding standard error ${\overset{ˆ}{s}}_{j}$ for each j=1,…,T. This problem—estimating a logodds ratio and its standard error—has been well studied (e.g., Gart and Zweifel, 1967). The main challenge is in dealing satisfactorily with cases where the maximum likelihood estimator for $\alpha_{j}$ is infinite. We use estimates based on results from Gart and Zweifel (1967); see Appendix B

Applying ASH to the estimates ${\overset{ˆ}{\alpha }}_{j}$ and standard errors ${\overset{ˆ}{s}}_{j}$ yields a posterior distribution for each $\alpha_{j}$. The simplest way to convert this to an estimate of the mean, μ, is to estimate $\alpha_{j}$ by its posterior mean, then reverse the above reparameterization. (Recovering μ also requires an estimate of $\mu_{1:T}$. We use the maximum-likelihood estimate, which is ${\overset{ˆ}{\mu }}_{1:T}=y_{1}+\cdots +y_{T}$.) The resulting estimate of each $\mu_{t}$ is the exponential of the posterior mean for $log\;\mu_{t}$ (because each $log\;\mu_{t}$ is a linear combination of the $\alpha_{j}$ ‘s). Alternatively, we can estimate each $\mu_{t}$ by approximating its posterior mean using the delta method; see Appendix B Both methods are implemented in our software. For the results below, we use the delta method because it is more comparable with previous approaches that estimate μ on the original scale rather than the logarithmic scale.

### 3.3. Practical Implementation

In practice, we follow these additional steps, guided by prior work, to improve performance and reduce effort.

Rather than use a single wavelet transform, we use the “translation invariant” wavelet transform (also called the “non-decimated” wavelet transform), which averages results over all T possible rotations of the data (effectively treating the observations as coming from a circle, rather than a line). Although not always necessary, this is a standard trick to reduce artifacts that can occur near discontinuities in the underlying signal, and can often improve performance (e.g., Coifman and Donoho, 1995). Implementation of the translation invariant wavelet transform for the Poisson model is described in Appendix B.3

The non-decimated wavelet transform yields T WCs at each of the $J={log}_{2}\;(T)$ resolution levels. We follow Johnstone and Silverman (2005b) in applying EB shrinkage separately to the WCs at each resolution level so that a different distribution g is estimated at each resolution. This is important because sparsity in the WCs ${\tilde{\mu }}_{j}$ will likely vary with resolution, and therefore the amount of shrinkage to apply should also be resolution-specific.

Although we have presented the DWT as a matrix-vector multiplication, which would naively take $O\left(T^{2}\right)$ operations, in practice there exist more efficient algorithms taking only $O\left(T{log}_{2}\;T\right)$ operations (Beylkin, 1992; Coifman and Donoho, 1995). These are implemented in the R package wavethresh (Nason, 2016), for example.

## 4. Results

We have conducted a wide range of numerical experiments to compare SMASH against the existing methods for wavelet-based signal denoising. Before presenting the results from these experiments (Section 4.2), we first illustrate the features of SMASH in a small example (Section 4.1). In Section 4.3, we present two applications of SMASH.

We have developed a companion repository containing all the source code (R and MATLAB), as well as the data used to generate the results, figures and tables presented below (Xing et al., 2021). This resource includes a “Shiny” Web app (Chang et al., 2018) for browsing the full results of the the simulation study (Section 4.2.1).

### 4.1. Illustration

**Figure 1:**
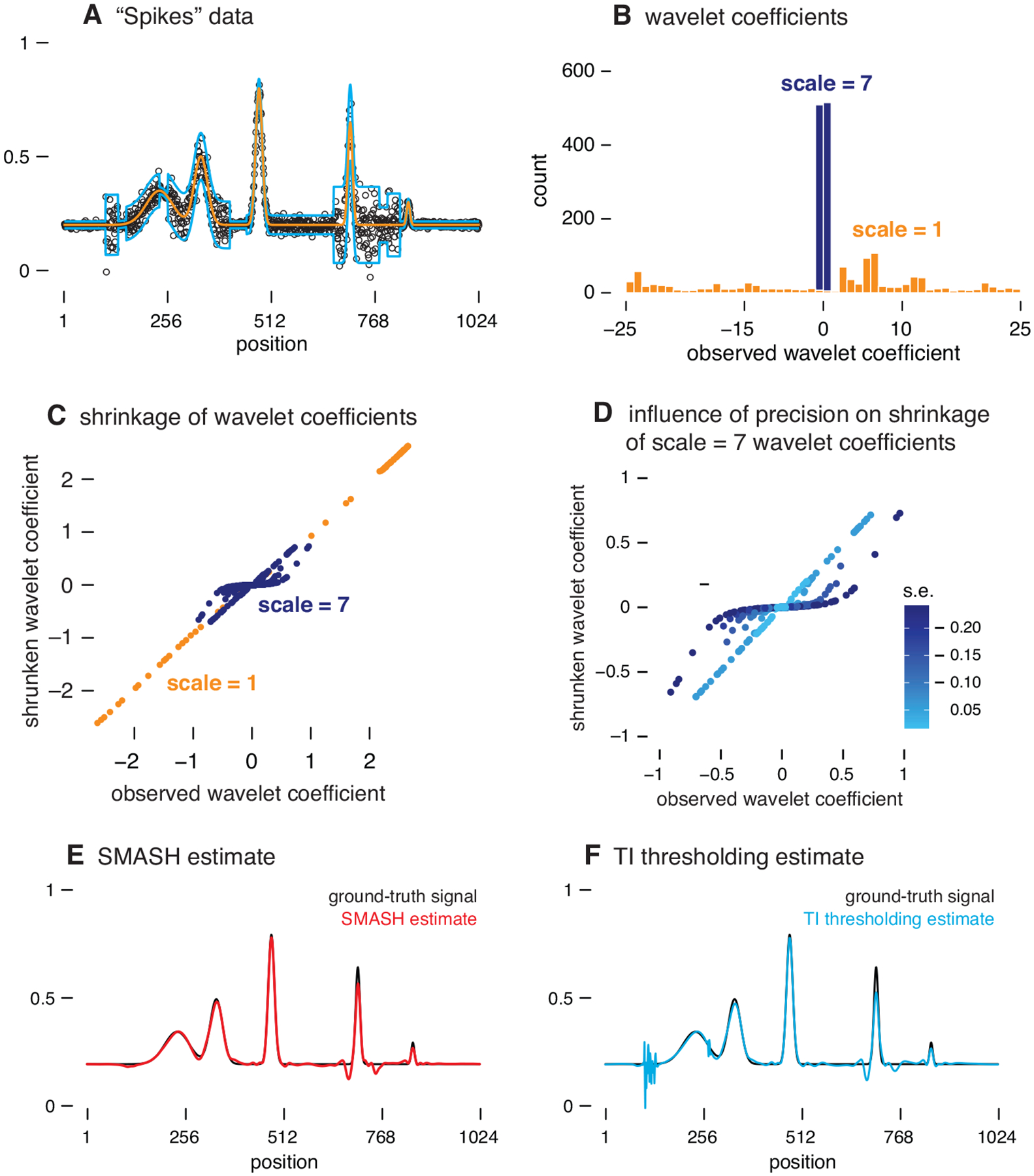
Illustration of SMASH, which is based on the empirical Bayes shrinkage method, ASH. Panel A shows the “Spikes” mean function (orange line) and “Clipped Blocks” variance function (light blue lines; \pm 2 standard deviations are shown) used to simulate the data. The simulated data points $y={\left(y_{1},\ldots ,y_{T}\right)}^{⊤}$ are shown as **black circles** (०). Panel B contrasts the distributions of the simulated wavelet coefficients (WCs), ${\tilde{y}}_{j}$, at a coarser scale (scale = 1, orange) and finer scale (scale = 7, dark blue). Note that the scale = 7 WCs are more concentrated near zero because the signal is smoother at this scale. Panel C compares the ASH shrinkage at these two scales; the scale = 7 WCs are strongly shrunk toward zero, whereas the scale = 1 WCs are not shrunk nearly as much. ASH infers that the scale = 7 WCs are heavily concentrated around zero, and consequently ASH shrinks them more strongly. Panel D illustrates that ASH shrinks WCs differently depending on their precision; specifically, the scale = 7 WCs that are less precise—that is, higher standard error (s.e.)—are shrunk more strongly toward zero. Panels E and F show the signals, $\mu ={\left(\mu_{1},\ldots ,\mu_{T}\right)}^{⊤}$, reconstructed by SMASH (red) and translation-invariant (TI) thresholding (Gao 1997; light blue), compared against the true mean function (**black**). The TI thresholding estimate shows notable artifacts. This example is implemented by the “Spikes” demo in the companion source code repository.

Figure 1 illustrates the key features of SMASH applied to smoothing a heteroskedastic Gaussian signal. The data in this example were simulated with a mean and variance that are both spatially structured (Figure 1, Panel A).

The first step in SMASH is to compute the WCs at different scales by applying the DWT. Each observed wavelet coefficient, ${\tilde{y}}_{j}$, can be viewed as a noisy estimate of some unknown “true” wavelet coefficient, ${\tilde{\mu }}_{j}$. These wavelet coefficients ${\tilde{\mu }}_{j}$ will be estimated using empirical Bayes shrinkage (14). Each WC, ${\tilde{y}}_{j}$, is associated with a standard error, $\omega_{j}^{2}$, that depends on the simulated variance of the data (15).

A key idea behind wavelet denoising is to “shrink” the observed WCs towards zero, resulting in an estimate of the mean that is smoother than if it were based solely on the observed data. A crucial question is, of course, how much to shrink. The ASH shrinkage method, which underlies SMASH, adapts the amount of shrinkage to the data in two distinct ways. If many observed WCs ${\tilde{y}}_{j}$ are large at a particular scale (that is, compared with their standard errors), ASH infers that, at this scale, many of the true WCs ${\tilde{\mu }}_{j}$ must also be large—that is, the estimated distribution g(2-3) has a long tail. Consequently, ASH shrinks less at this scale than at scales where few observed WCs are large, in which case the estimated g will have a short tail. This is illustrated in Figure 1, Panels B and C. At scale =1, many observed WCs are large (Panel B), so little shrinkage is applied to these WCs (Panel C). By contrast, at scale =7, few observed WCs are large (Panel B), and therefore stronger shrinkage is applied (Panel C). This adaptive feature is also characteristic of other EB shrinkage methods, but the family of unimodal distributions underlying ASH is more flexible, increasing its potential to adapt to different contexts. Second, because the posterior distribution (5) incorporates the standard error of each observation, shrinkage is adaptive to the standard error; at a given scale, WCs ${\tilde{y}}_{j}$ with larger standard errors $\omega_{j}$ are shrunk more strongly than WCs with small standard errors. This is illustrated in Panel D. (In this example, the standard errors vary among WCs due to the spatially structured variance of the simulated data.)

The end result is that (i) data that are consistent with a smooth signal are smoothed more strongly, and (ii) smoothing is stronger in areas of the signal with greater variance. The smoothed signal from SMASH (Figure 1, Panel E) is noticeably more accurate than the signal estimated using TI thresholding in Panel F (in which the variance is estimated using the “median absolute deviation,” or RMAD, method of Gao 1997).

We return to this simulation scenario in Section 4.2.1, where we compare the performance of SMASH against signal denoising methods more systematically in many simulated data sets.

### 4.2. Simulations

We investigated the signal denoising performance of SMASH against existing approaches in data sets simulated from Gaussian and Poisson distributions.

#### 4.2.1. Gaussian Mean Estimation

In our first set of simulations, we ran different methods for estimating a spatially structured mean from Gaussian-distributed observations, and assessed accuracy of the estimates. Our simulation study was modeled after Antoniadis et al. (2001). Specifically, we used many of the same test functions (7 mean functions, 5 variance functions) and two different signal-to-noise ratios, 1 and 3 (Figures 9 and 10). For each combination of simulation settings, we simulated 100 data sets, each with a signal of length T=1,024, and applied the signal denoising methods to each of the simulated data sets. In all cases, we ran three variations of SMASH: when the variance function was estimated, allowing for heteroskedasticity; when variance was estimated, assuming homoskedasticity; and when SMASH was provided with the ground-truth variance function, which could be viewed as a “gold standard.” We compared these SMASH variants against the Translation Invariant (TI) thresholding method (Coifman and Donoho, 1995), which was one of the methods shown that performed best in Antoniadis et al. (2001). We also compared against the empirical Bayes shrinkage procedure, “EbayesThresh” (Johnstone and Silverman, 2005a). For all results shown in the figures and tables below, the methods used the Symmlet8 wavelet basis (Daubechies, 1992). To assess performance of the methods, we report the mean integrated squared error (MISE), which summarizes the difference between the ground-truth and estimated mean signal (Nason, 1996). R and MATLAB scripts implementing these comparisons, as well as the results generated using these scripts, are provided in the companion repository (Xing et al., 2021).

**Figure 2:**
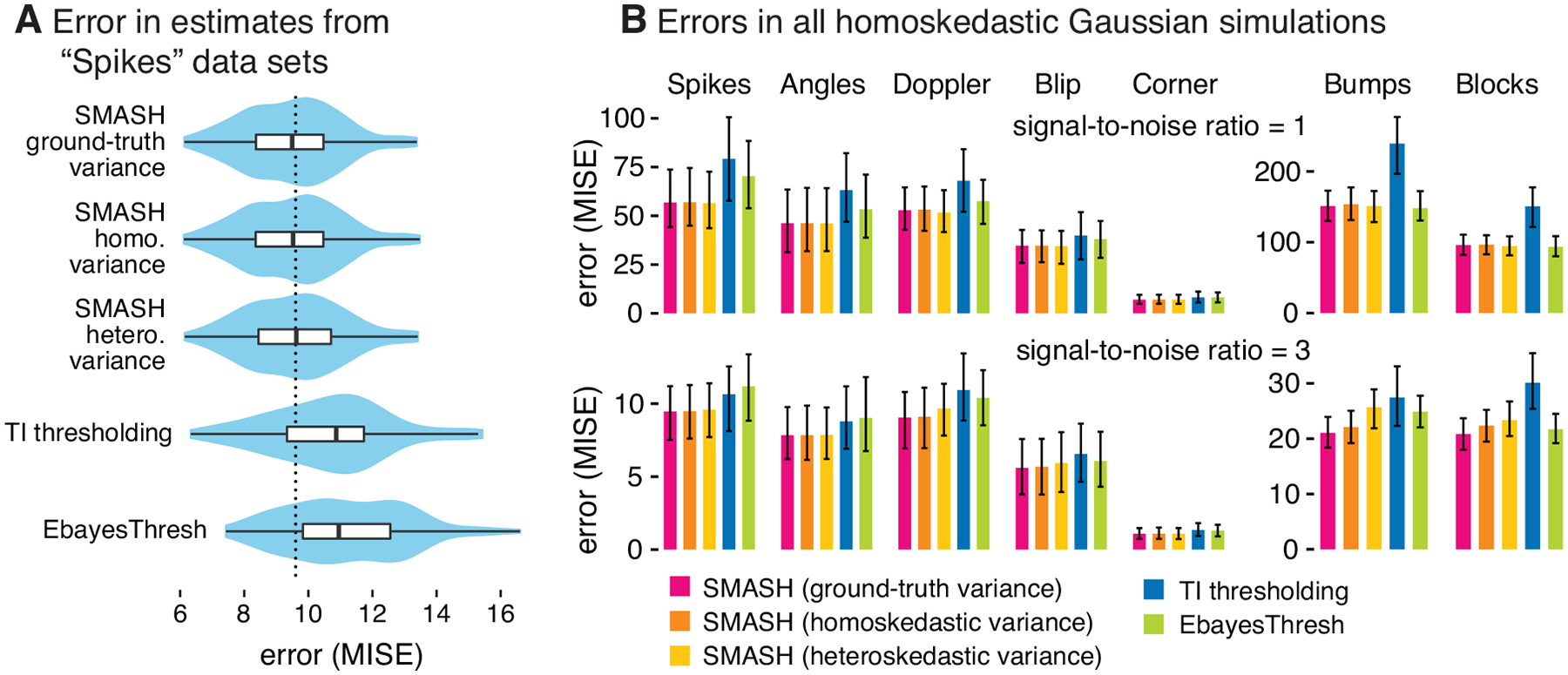
Accuracy of mean signal estimates applied to data sets simulated with homoskedastic Gaussian noise. Panel A shows violin plots (and inset boxplots) summarizing the error (MISE) of the estimates in the “Spikes” simulation scenario with constant variance and a signal-to-noise ratio of 3. In Panel B, bars give the average error (MISE) in the mean estimates across all simulations; error bars show the 10% and 90% quantiles. The functions used to simulate data sets in each scenario (corresponding to columns of Panel B) are shown in Figure 9. Methods compared are: SMASH with homoskedastic variances; SMASH allowing for heteroskedastic variances; SMASH when the ground-truth variance is provided; TI thresholding; and EbayesThresh. (Note that both TI thresholding and EbayesThresh assume homoskedastic variances.) In the “Spikes” scenario (Panel A), all variants of SMASH outperformed TI thresholding and EbayesThresh; overall (Panel B), SMASH consistently performed as well as or better than the other methods.

We first focus on the simulations with homoskedastic variance. Figure 2 compares the performance of each of the methods in this setting. In the “Spikes” scenario (Panel A), all three variants of SMASH outperformed EbayesThresh and TI. Further, the three SMASH variants yielded estimates of comparable accuracy. This illustrates that allowing for heteroskedasticity when the truth is homoskedastic can sometimes be done with little or no loss of accuracy. Most of the other simulation settings with homoskedastic variance show similar trends (Figure 2, Panel B). For the most difficult settings—”Bumps” and “Blocks” with a signal-to-noise ratio of 1—EbayesThresh achieved similar accuracy to SMASH, whereas TI thresholding performed much worse.

Next, we examine the performance of the same methods in data sets simulated with heteroskedastic errors. Since the performance of the TI thresholding method with homoskedastic variances was consistently poor (see the interactive plot), we considered three different ways to allow for heteroskedastic variances in TI thresholding: providing the ground-truth variance; estimating variances using SMASH; and estimating variances using the extended RMAD method of Gao (1997) (henceforth “RMAD” for short).

**Figure 3:**
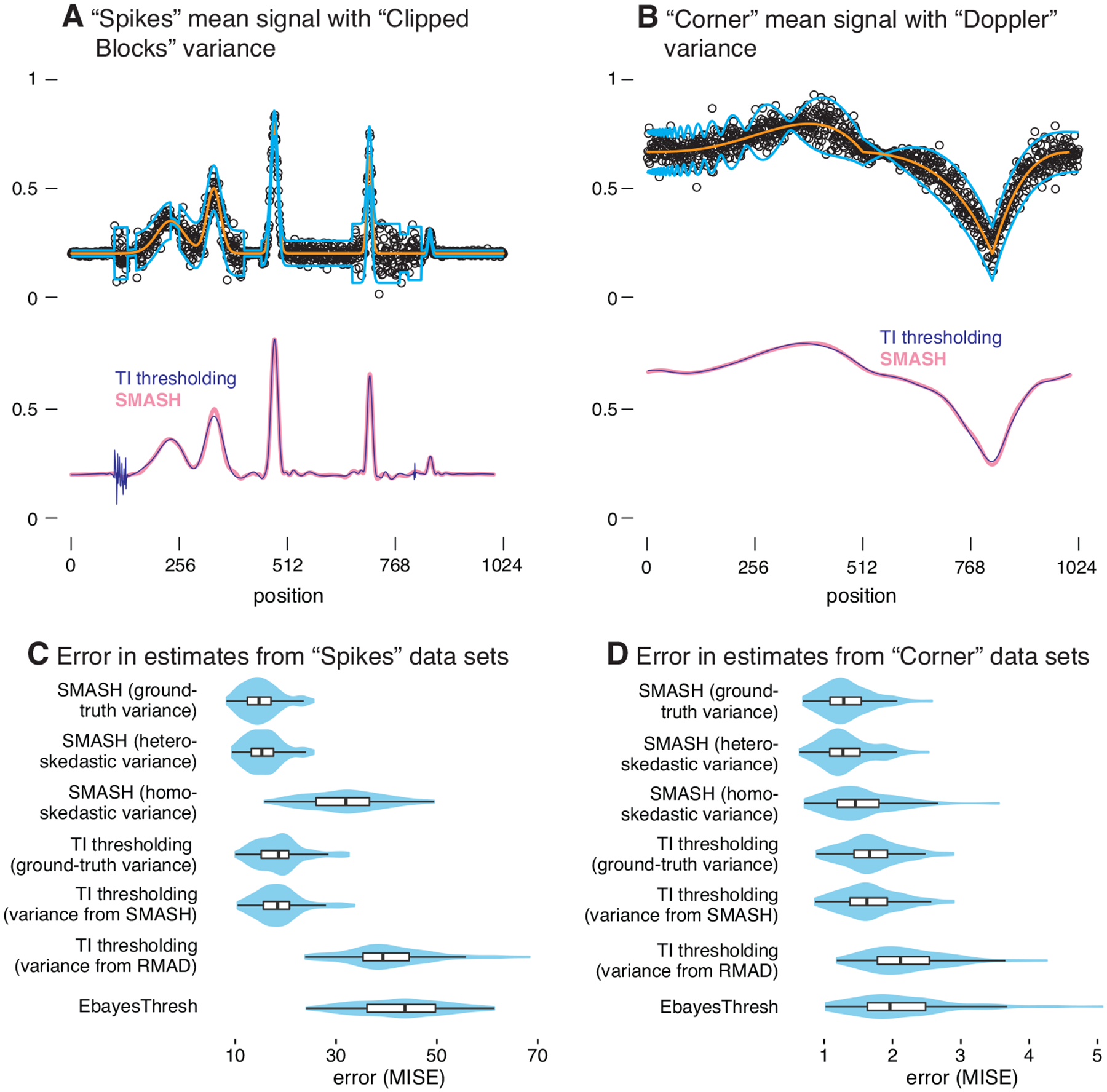
Illustration of signal denoising methods applied to Gaussian data sets simulated with heteroskedastic errors. Panels A and B depict the mean signals (orange lines) and variance functions (light blue lines, showing ±2 standard deviations) used to simulate the data. An example simulated data set is shown in each case (**black circles**, ∘). The signals recovered by TI thresholding with RMAD variance estimates (dark blue line) and SMASH with estimated heteroskedastic variances (pink line) are also shown for these two data sets. Panels C and D give violin plots (and inset boxplots) summarizing the error (MISE) in the mean estimates. Methods compared are: SMASH with homoskedastic variances, with the ground-truth variances, and allowing for heteroskedastic variances; TI thresholding with SMASH-estimated variances, with RMAD-estimated variances, and with ground-truth variances; and EbayesThresh.

**Figure 4:**
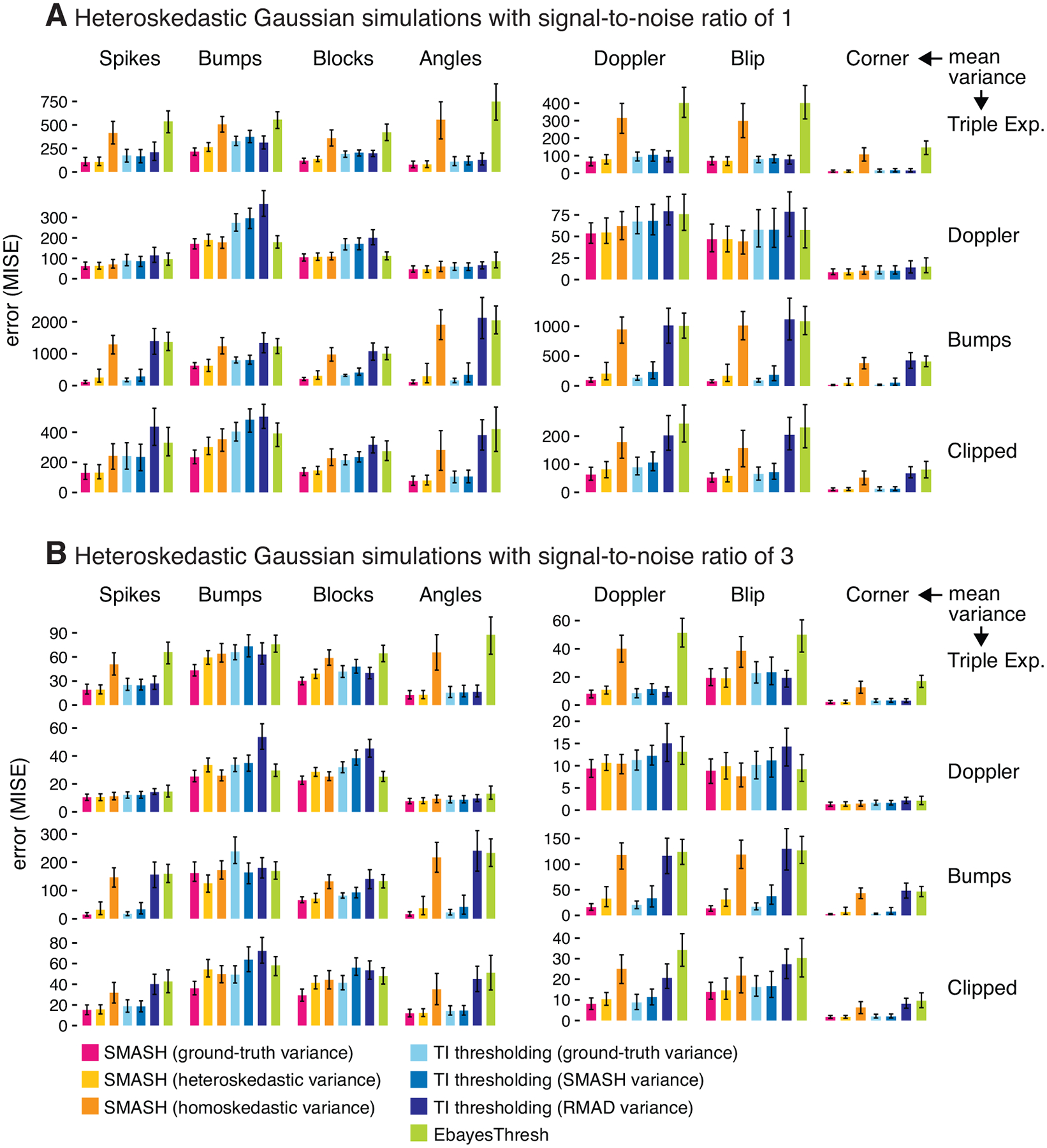
Comparison of signal denoising methods in Gaussian data sets simulated with heteroskedastic error, with a signal-to-noise ratio of 1 (Panel A) and 3 (Panel B). Bars give the average error (MISE) in the mean estimates across all simulations; error bars show the 10% and 90% quantiles. Each scenario is defined by a combination of the mean function (columns) and variance function (rows) used to simulate the data (these functions are depicted in Figures 9 and 10). In each scenario, 100 data sets were simulated. Methods compared are: three variants of SMASH (with homoskedastic variances, ground-truth variances, and allowing for heteroskedastic variances); three variants of TI thresholding (with SMASH-estimated variances, RMAD-estimated variances, and ground-truth variances); and EbayesThresh.

Figure 3 provides a detailed view of performance on data sets simulated with a signal-to-noise ratio of 3: the “Spikes” mean function with the “Clipped Blocks” variance function (Figure 3, Panels A, C); and the “Corner” mean function with the “Doppler” variance function (Figure 3, Panels B, D). Figure 4 summarizes the results from all simulations. The results of all these simulations can be explored interactively in the Shiny plot included in the companion repository.

Allowing for heteroskedasticity in SMASH substantially improved its accuracy in all settings; compare the yellow and orange bars in Figure 4. Further, in nearly all settings SMASH with estimated heteroskedastic variance generally performed at least as well as, and often much better than, EbayesThresh and all TI thresholding variants. While the improvements were greatest in data sets simulated with sudden, large changes to the variance (“Bumps” and “Clipped Blocks” variance functions), what is perhaps more remarkable is that SMASH provided consistently competitive performance in all settings.

We comment now on some other key trends arising from the results shown in Figure 4. First, SMASH with estimated heteroskedastic variance often achieved comparable accuracy to SMASH with the ground-truth variance. However, some variance functions are harder to estimate than others (e.g., the “Bumps” and “Clipped Blocks” functions), and in such cases providing the method with the ground-truth variance usually improved accuracy. Second, EbayesThresh generally performed much less competitively here than in the homoskedastic setting, which highlights the importance of accounting for heteroskedasticity. The most extreme example of this is in simulations with the “Triple Exponential” variance test function, which has large changes in variance, but the changes are gradual enough that estimating the variance can be done accurately. Consistent with the results in Figure 2, SMASH with homoskedastic variance consistently performed better than, or at least as well as, EbayesThresh.

Finally, TI thresholding generally performed better when used with the SMASH variance estimate than with the RMAD variance estimate. The largest differences in performance were in simulations with more abrupt changes to variances; indeed, the RMAD estimates performed well in simulations with the smoother “Triple Exponential” variance function. This suggests that the RMAD method works best in settings where the variance changes gradually.

#### 4.2.2. GaUSSIAN VARIANCE ESTIMATION

An unusual feature of SMASH is that it performs joint mean and variance estimation. We found no R packages for doing this in the wavelet context. We only found one publication on wavelet-based variance estimation, Cai and Wang (2008), in which a wavelet thresholding approach is applied to first-order differences in the data. Non-wavelet-based approaches related to this work include a method by Fan and Yao (1998), which estimates the variance by smoothing the squared residuals using local polynomial smoothing; Brown and Levine (2007), which uses difference-based kernel estimators; and Menictas and Wand (2015), which introduces a Mean Field Variational Bayes (MFVB) method for joint mean and variance estimation. In all cases, we could not find publicly available software implementations of these methods. However, we did receive code implementing MFVB via correspondence with M. Menictas, and we used this code in our comparisons.

**Figure 5:**
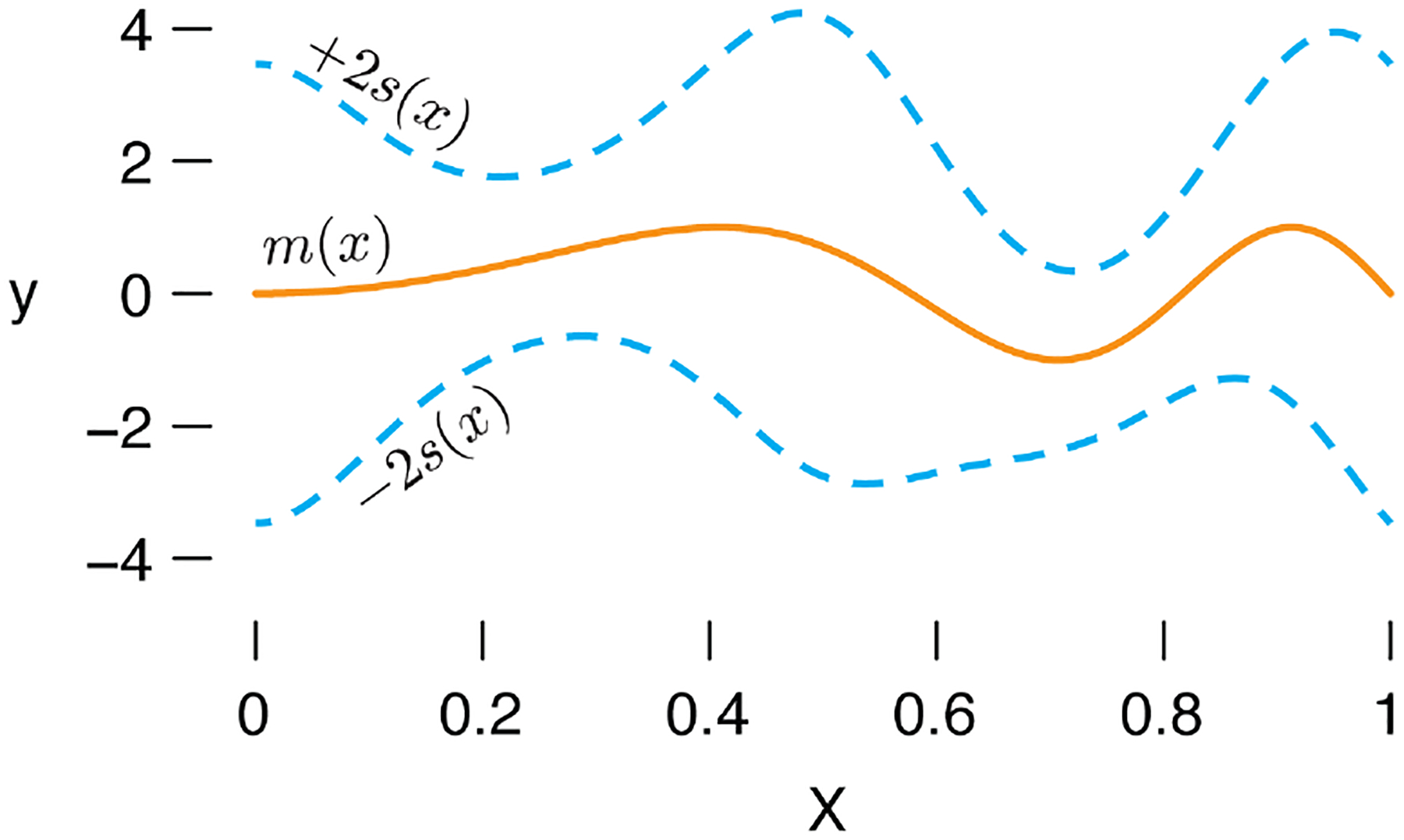
The mean function, m(x) (orange lines), and ±2 standard deviations, s(x) (dashed, light blue lines), used to simulate the data sets for comparing SMASH and MFVB. The same mean and standard deviation functions were used for “Scenario A” from Figure 5 in Menictas and Wand (2015).

The MFVB method is based on penalized splines, so it is not well suited to many of the standard test functions in the wavelet literature—these test functions often contain “spiky” local features that are not well captured by splines. Therefore, for fair comparison, we applied SMASH and MFVB to smooth mean and variance functions; specifically, we generated data following “Scenario A” in Figure 5 from Menictas and Wand (2015) using scripts kindly provided by M. Menictas. The mean and variance functions are shown in Figure 5

We evaluated SMASH and MFVB in two scenarios. In the first scenario, we simulated unevenly spaced data points: we independently generated T=500 pairs $\left(X_{t},y_{t}\right)$, with $X_{t}\sim \text{Uniform}(0,1)$ and $y_{t}|X_{t}=x_{t}\sim N\left(m\left(x_{t}\right),s{\left(x_{t}\right)}^{2}\right)$, in which m(⋅) and s(⋅) denote the mean and standard deviation functions shown in Figure 5. To assess accuracy, we computed the mean of the squared errors (MSE) evaluated at 201 equally spaced points within [min(X),max(X)], where min(X) and max(X) are the smallest and largest values of $X=\left(X_{1},\ldots ,X_{T}\right)$, respectively. We computed the MSE separately for estimates of the mean and standard deviation. For both SMASH and MFVB, estimates of the mean and variance at each of the 201 equally spaced points were obtained by a simple linear interpolation between the available estimates at the two nearest flanking data points.

In this scenario, SMASH could not be immediately applied to the simulated data because the points were not equally spaced, and the number of data points was not a power of 2. To address the first issue, we followed the common practice of treating the observations as if they were evenly spaced (see Sardy et al., 1999, for discussion). To deal with the second issue, we borrowed a standard trick used in the wavelet literature; first, we reflected the data about the right edge and extracted the first $2^{\left\lfloor {log}_{2}\;(2T)\right\rfloor }=512$ data points, so that the number of data points in the new data set was a power of 2, and so that the mean curve was continuous at the right edge of the original data. Further, to ensure that the input to SMASH was periodic, we reflected the transformed data set about its right edge, so that the final transformed signal was of length 1,024. After running SMASH, the estimates of the mean and variance functions were extracted from the first T=500 positions.

In the second scenario, we simulated evenly spaced data points; we independently generated T=1,024 pairs $\left(X_{t},y_{t}\right)$, with the $X_{t}$ ‘s equally spaced on [0, 1]. Performance was evaluated separately for the mean and standard deviation as the mean of the MSEs evaluated at each of the locations, t=1,…,T.

For each scenario, we simulated 100 data sets. These experiments are implemented in the “Gaussian variance estimation” analysis in the companion repository.

Table 1 shows, for each scenario, the mean error (MSE) in the estimated mean and standard deviation, averaged over the 100 independent simulations. Despite the fact that these simulation scenarios, particularly Scenario 1, seem better suited to MFVB than SMASH, SMASH performs comparably or better than MFVB for both mean and variance estimation.

#### 4.2.3. POISSON DATA

In our final set of simulations, we assessed the ability of different methods to reconstruct a spatially structured signal from Poisson-distributed data. Similar to the Gaussian simulations, we generated data sets using a variety of test functions and intensity ranges. Specifically, we considered 6 test functions from Besbeas et al. (2004); Fryzlewicz and Nason (2004); Timmermann and Nowak (1999) (see Figure 11), and defined μ by rescaling the test function so that the smallest intensity was x and the largest intensity was y, with (x,y) set to either (1/100,3),(1/8,8) or (1/128,128). For each combination of test function and intensity range, we simulated 100 data sets, each with a signal of length T=1,024. We measured the accuracy of the estimates using the mean integrated squared error (MISE), as above.

We compared SMASH against the Bayesian multiscale model (BMSM) and Haar-Fisz (HF) methods. BMSM is an empirical Bayes method, like SMASH, but with a less flexible prior distribution on the multi-scale coefficients (Kolaczyk, 1999). The Haar-Fisz method (Fryzlewicz and Nason, 2004) performs a transformation of the Poisson counts, then applies Gaussian wavelet methods to the transformed data. There are many choices for Gaussian wavelet methods, and the performance of the HF method is strongly dependent on which Gaussian wavelet method is chosen, with different choices being better for different data sets. We evaluated the performance of four variants of the HF method, with details given in Appendix C. Based on our empirical comparisons, we found that the HF method with Gaussian denoising implemented using the non-decimated wavelet transform and universal thresholding (Donoho and Johnstone, 1994), and with a fixed noise level, yielded the best estimates in most simulation scenarios, so in our results we report results from the HF method with these settings.

**Figure 6:**
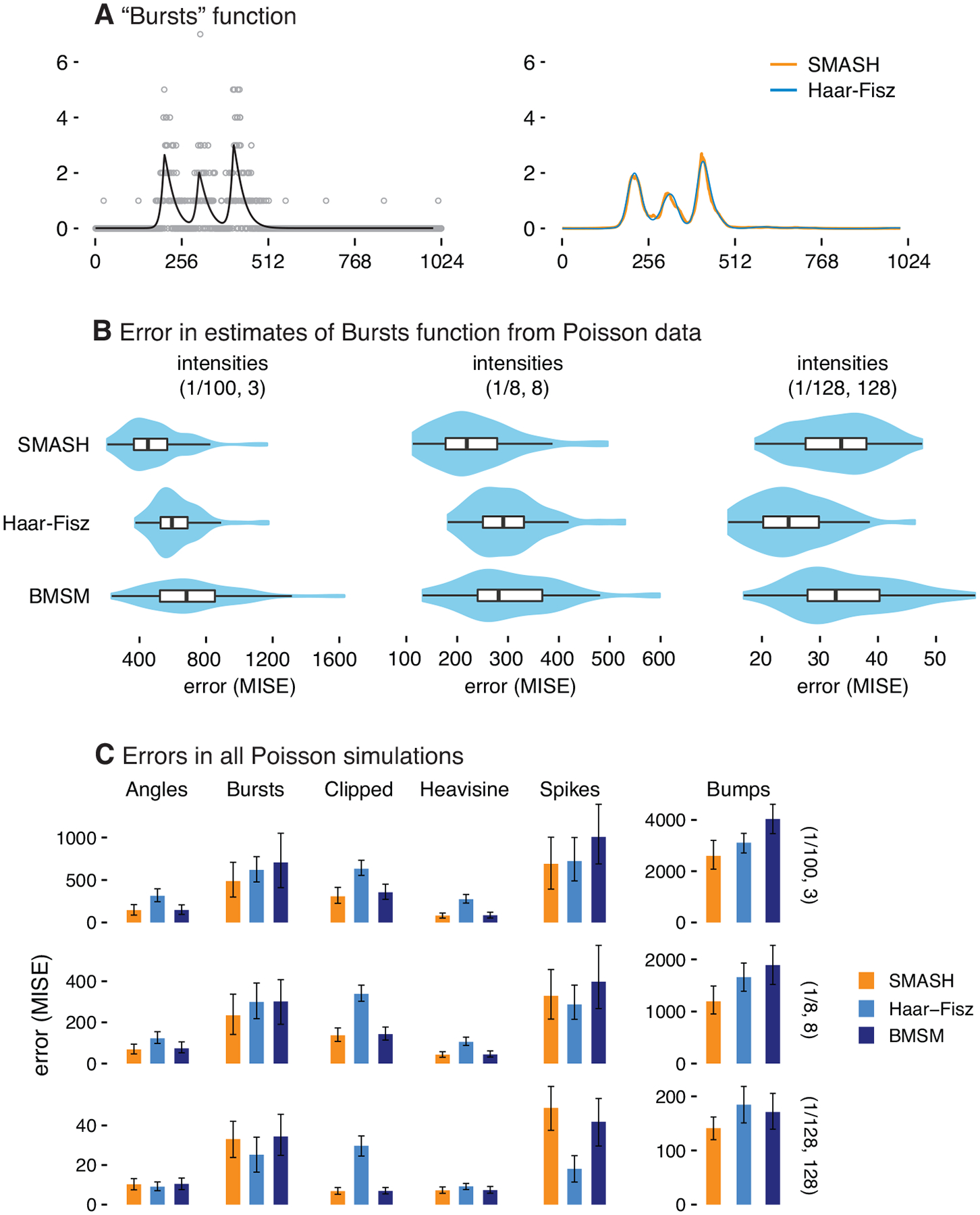
Comparison of signal noising methods in Poisson data sets simulated with a variety of test functions and intensity ranges. For illustration, Panel A shows the “Bursts” test function (black line) and an example data set (gray circles) which was simulated at the (1/100, 3) range of intensities. The reconstructed signals (SMASH, orange line; HF, light blue line) for this example data set are also shown. Panel C summarizes the error (MISE) in the mean estimates for all simulations, and Panel B gives a more detailed summary of the results from the “Bursts” simulations. Error bars show the 10% and 90% quantiles. The test functions used to simulate the data sets are shown in Figure 11. (Note the results for the “Bumps” simulations are plotted at a different scale because the MISE is much higher in these simulations.) For each of the scenarios, a total of 100 data sets were simulated at each intensity range, (1/100,3),(1/8,8) and (1/128,128). Methods compared are SMASH, BMSM (Kolaczyk, 1999), and the Haar-Fisz method (Fryzlewicz and Nason, 2004) with a non-decimated wavelet transform and universal thresholding.

The results of these simulations are summarized in Figure 6 (additional figures and tables with more detailed results for all simulation settings are included in the companion repository). In almost all simulation scenarios, SMASH performed as well or better than the HF and BMSM methods, with the greatest gains occurring in the more challenging, lower intensity scenarios. The only scenario where SMASH was clearly outperformed by another method was in the spikes scenario with a high intensity range, where the HF method outperformed the other methods. Comparing BMSM with HF, neither dominated the other; sometimes the BMSM method was better, whereas in other settings the HF method was better. As noted above, the HF transform can be used in a variety of ways, so results here should be viewed only as a guide to potential performance.

One practical limitation of the HF transform is that, to achieve translation invariance, the transform has to be done explicitly for each shift of the data; the tricks usually used to do this efficiently (Coifman and Donoho, 1995) do not work here. Thus, making HF fully translation invariant increases computation by a factor of T, rather than the factor of log (T) as for the other methods. We followed the advice of Fryzlewicz and Nason (2004) and reduced the computational burden by averaging over 50 shifts of the data rather than T shifts. With this approximation, the HF method was slower than the other methods, but not by a lot. A direct comparison of computational efficiency between SMASH and BMSM is difficult as they are coded in different programming environments. Nevertheless, similarities between the two methods suggest that they should have similar computational cost. In our simulations, the runtime of all three methods was typically a few seconds or less per data set.

### 4.3. Illustrative Applications

In the experiments above, we showed that SMASH is accurate for denoising signals in simulated data sets, where the ground-truth signal is known. To further illuminate the features of SMASH, we used SMASH in two applications: analysis of motorcycle acceleration data, which has been studied in other wavelet denoising papers (Delouille et al., 2004; Silverman, 1985); and a problem from computational biology—calling “peaks” in chromatin immunoprecipitation sequencing (“ChIP-seq”) data (Robertson et al., 2007; Dunham et al., 2012).

#### 4.3.1. Motorcycle Acceleration Data

Here we demonstrate application of SMASH to the motorcycle acceleration data set from Silverman (1985). We chose this data set because it exhibits clear heteroskedacity, and because it has previously been found to be a challenging data set for wavelet methods; for example, Delouille et al. (2004) required *ad hoc* data processing steps, including filtering out the high-resolution wavelet coefficients, to produce an appealing fit.

The data consist of 133 observations measuring head acceleration from a simulated motorcycle accident that was used to test crash helmets. The dependent variable is acceleration (in g) and the independent variable is time (in ms). To deal with repeated measurements, we took the median of multiple acceleration measurements at each time point. As in the simulations of Section 4.2.2, we treated the data as if they were equally spaced. In this example, we compared SMASH to TI thresholding with RMAD variance estimates since this method tended to be competitive with SMASH in scenarios where changes to the variance were more gradual (Section 3.1). This example is implemented by the “Motorcycle Acceleration” analysis in the online companion code repository, which includes a comparison with other variants of SMASH and TI thresholding not shown here.

**Figure 7:**
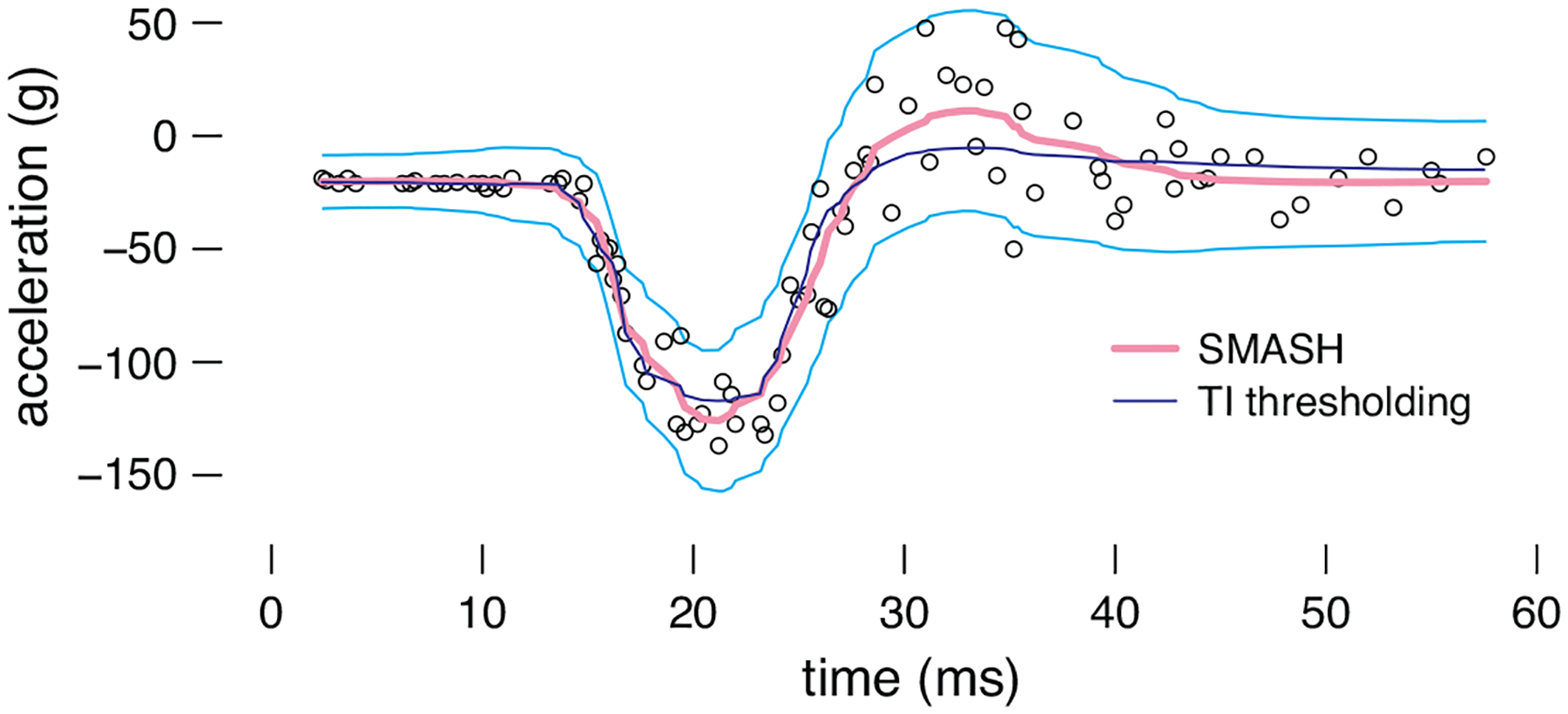
SMASH and TI thresholding applied to the motorcycle acceleration data (Silverman, 1985). The dark blue line shows the signal recovered by TI thresholding, with RMAD estimates of the variance, and the pink line shows the mean curve estimated by SMASH. The ±2 standard deviations estimated by SMASH are drawn as light blue lines. The data points are shown as **black circles** (∘).

The fitted SMASH and TI thresholding curves are shown in Figure 7. Without hand-tuning of any parameters, both methods provided a reasonable fit to the data. Visually, SMASH favoured a closer fit, whereas TI thresholding produced a slightly smoother curve. The nonparametric regression methods in Delouille et al. (2004) had more difficulty dealing with this data set (see Figure 11 of that paper).

#### 4.3.2. CHIP-SEQ DATA

Chromatin immunoprecipitation sequencing (“ChIP-seq”) is a widely used technique to measure transcription factor binding along the genome (Robertson et al., 2007). After preprocessing steps, the data are counts of sequencing reads mapped to locations along the genome. These counts can be treated as arising from an inhomogeneous Poisson process whose intensity at site b is related to the binding strength of the transcription factor near b (Anders and Huber, 2010; Marioni et al., 2008). Binding tends to be localized—the vast majority of counts are expected to be zero, with a small number of strong “peaks”. Identifying these peaks can help to identify regions where binding occurs, which is an important component to understanding gene regulation. Consequently, there are many methods for detecting “peaks” in ChIP-seq data (Wilbanks and Facciotti, 2010). Our goal here is to briefly describe how SMASH could provide an alternative approach to analyzing ChIP-seq data by first estimating the underlying intensity function. Once the intensity function has been estimated, “peaks” can be identified as regions where the estimated intensity function exceeds some predetermined threshold.

To illustrate the approach, we applied SMASH to a ChIP-seq data set collected as part of the ENCODE project (“Encyclopedia of DNA Elements”; Dunham et al., 2012). The data are ChIP-seq read counts at $2^{17}\approx 131,000$ locations (base-pair positions on chromosome 1). The signal is very sparse; over 98% of the read counts (128,999 out of 131,072 base-pair positions) are zero. The SMASH analysis consists of estimating the mean and variance of the underlying signal at these 2^17^ sites. For comparison, we also applied the Haar-Fisz method to these data (using the same settings used in Section 3.2). The SMASH and HF methods each took about 5 minutes to run on these data (MacBook Pro, 3.5 GHz Intel i7 multicore CPU, R 3.4.3, no multithreaded external BLAS/LAPACK libraries).

**Figure 8:**
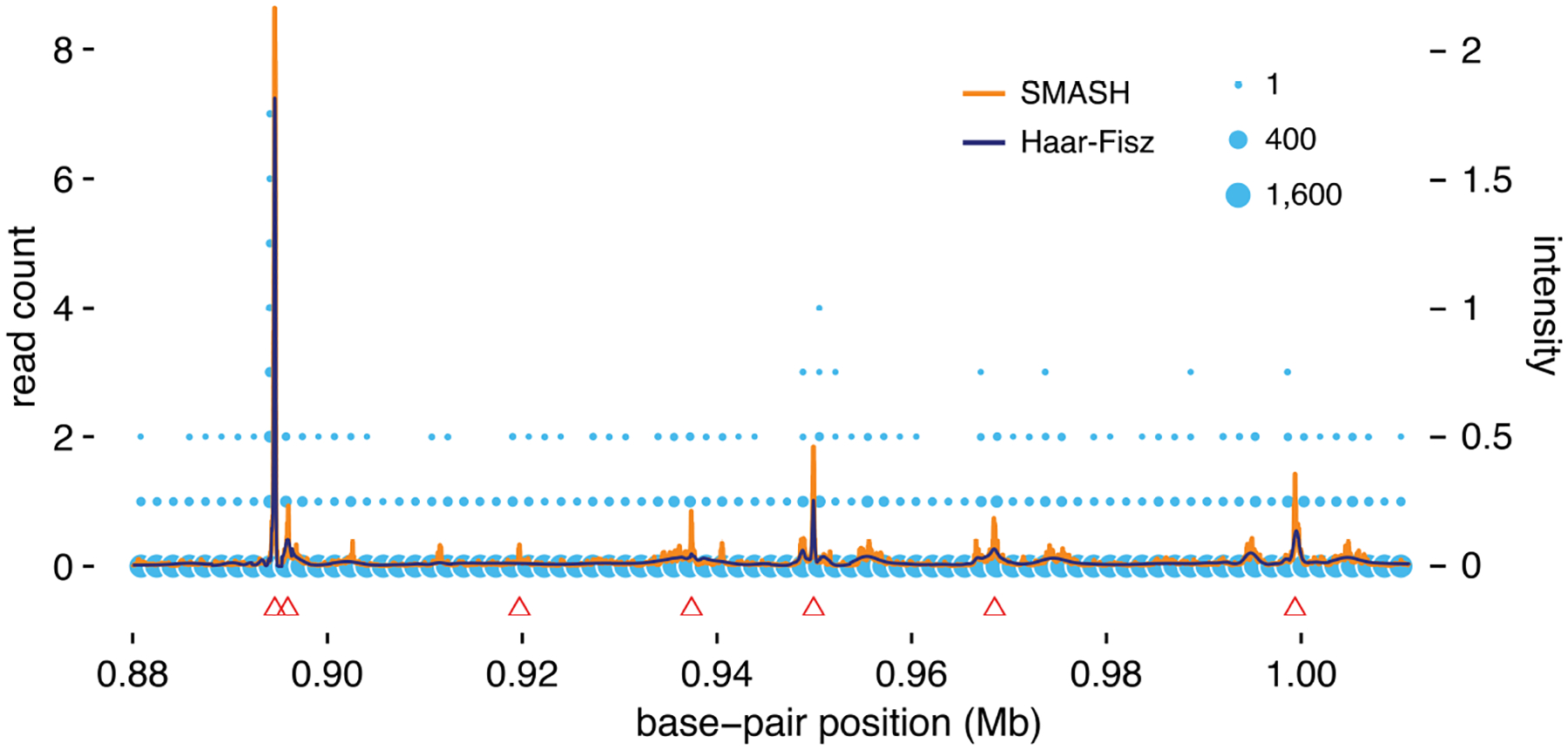
Illustration of SMASH for identifying peaks in ChIP-seq data. The data are ChIP-seq read counts for transcription factor YY1 in cell line GM12878 from the ENCODE project (“Encyclopedia of DNA Elements”; ENCODE Project Consortium, 2011; Dunham et al., 2012; Sloan et al., 2016; Gertz et al., 2013; Landt et al., 2012). Since this cell line has two ChIP-seq replicates (GEO accessions GSM803406 and GSM935482), the final counts were obtained by summing the read counts from both replicates. The region analyzed comprises base-pair positions 880,001–1,011,072 on chromosome 1, a region of $2^{17}\approx 131,000$ base-pairs in length. (Base-pair positions are based on human genome reference assembly 19, NCBI build 37.) Count data are depicted as light blue circles, with circle area scaled by the number of data points within each 1.6-kb bin. (Most counts are zero.) The orange line shows the intensity function μ estimated by SMASH, and the dark blue line shows the intensity function estimated by the HF method. MACS peaks (Zhang et al., 2008) are shown as red triangles (Δ). (These are the mean positions of the MACS peak intervals.) This example is implemented by the “Chipseq” analysis in the accompanying source code repository.

**Figure 9:**
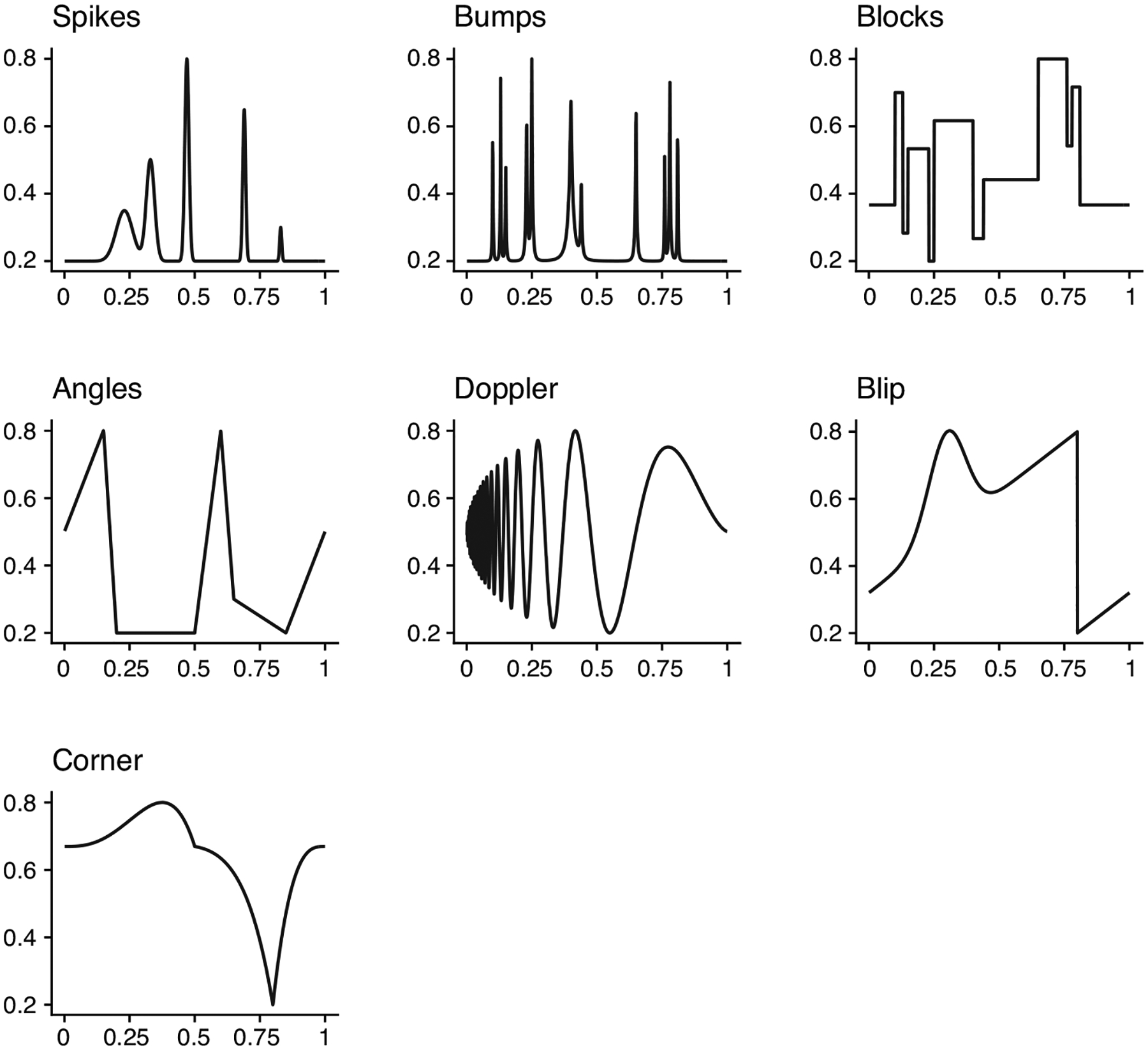
Mean functions used to simulate the Gaussian data sets.

**Figure 10:**
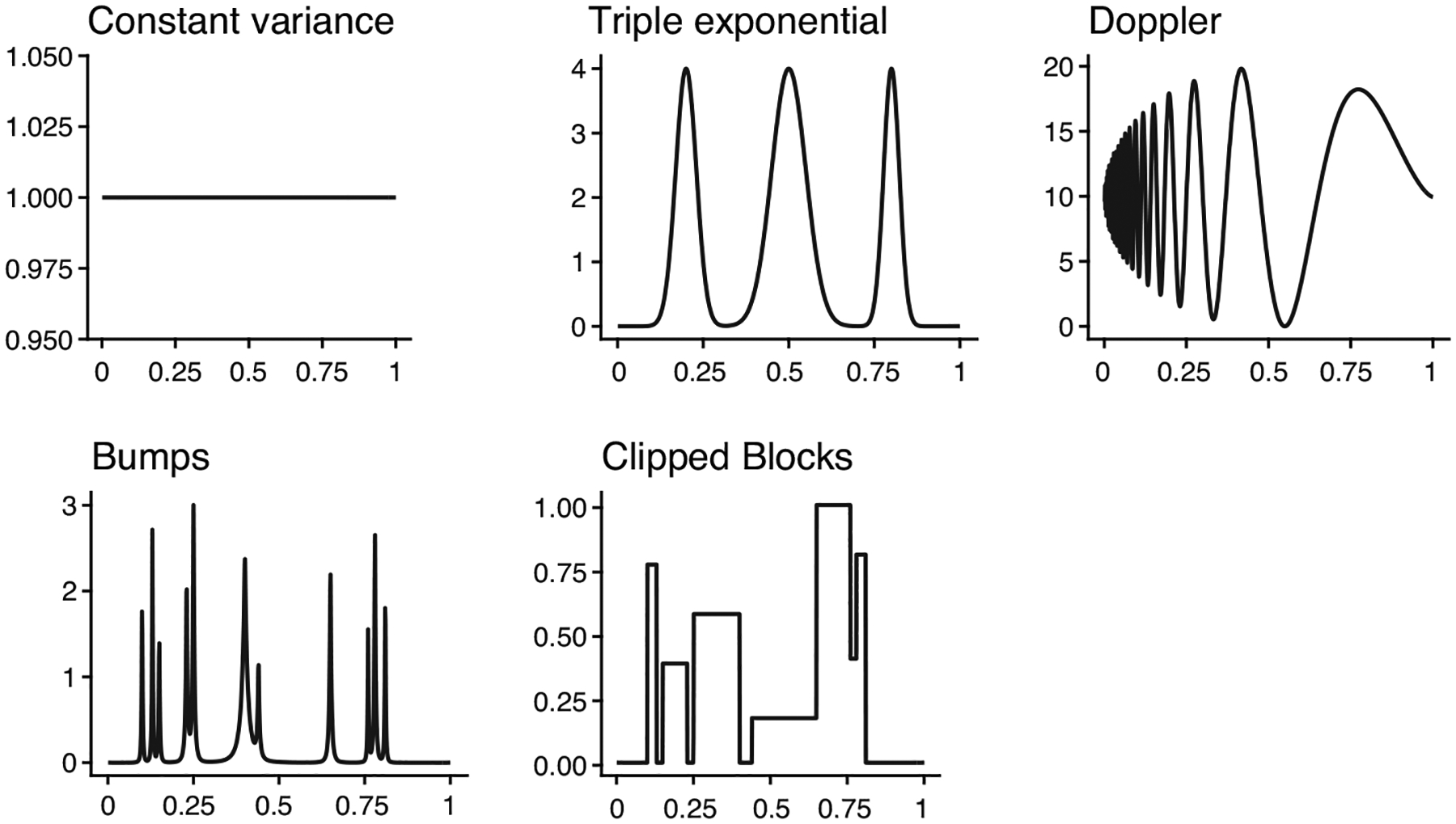
Variance functions used to simulate the Gaussian data sets. These functions were rescaled in the simulations to achieve the desired signal-to-noise ratio.

**Figure 11:**
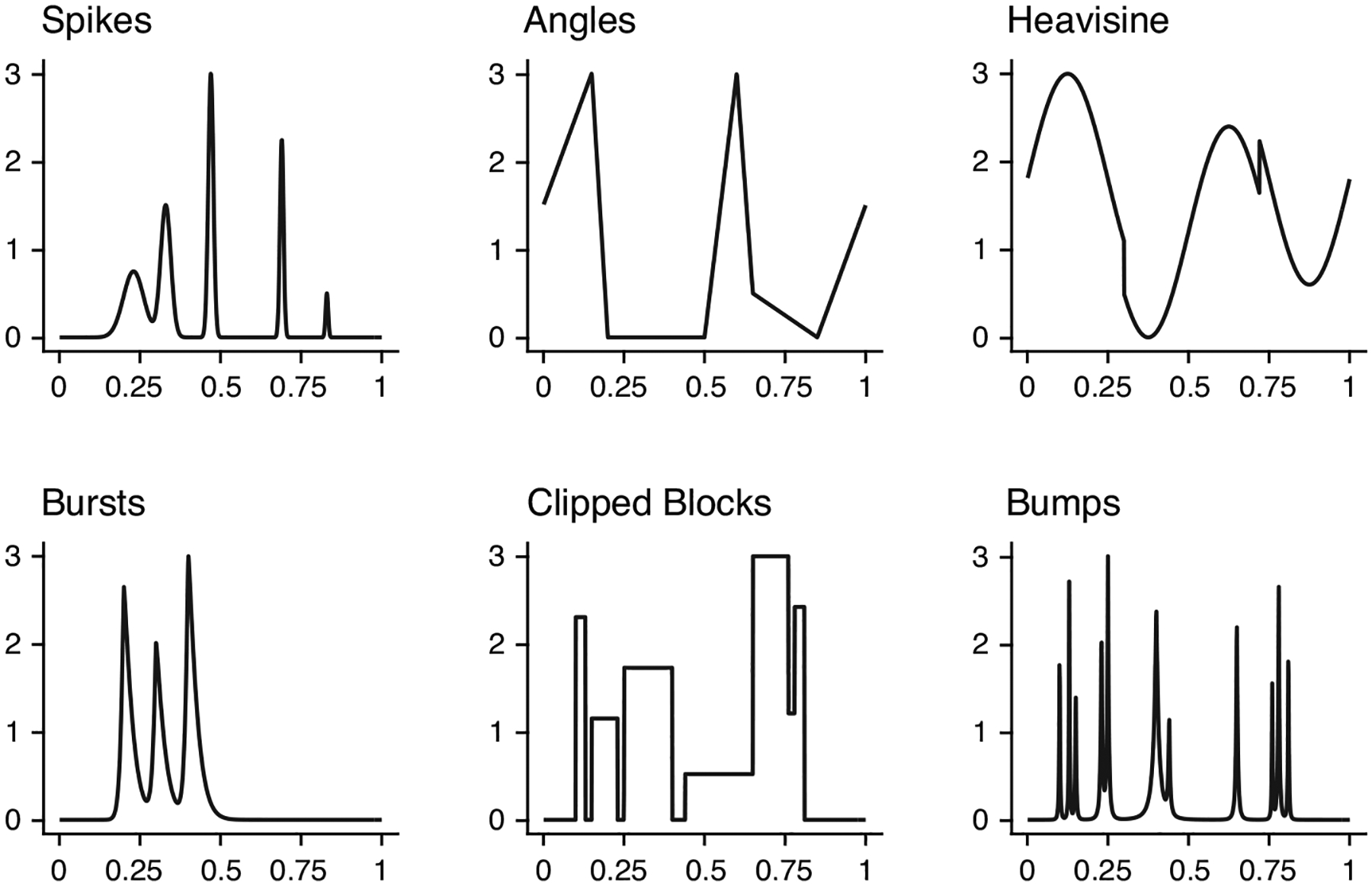
Intensity functions used to simulate the Poisson data sets.

The intensity functions μ estimated by SMASH and the HF method are shown in Figure 8. These estimates (the orange and dark blue lines) are overlaid with the ChIP-seq peaks (red triangles) identified by a widely used peak-calling software, MACS (Zhang et al., 2008). The locations with the strongest intensity estimates align closely with the peaks found by MACS. However, the HF method recovered fewer MACS peaks, and at a much reduced intensity. The SMASH estimates also suggest the presence of several additional weaker peaks not identified by MACS.

Reliable calling of peaks in ChIP-seq data is a multi-faceted problem, and a full assessment of the potential for SMASH to be applied to this problem lies outside the scope of this paper. Nonetheless, these results suggest that this approach could be worth pursuing. One benefit of our multi-scale Poisson approach is that it deals well with a range of intensity functions, and could perform well even in settings where peaks are broad or not well-defined. By contrast, the performance of different peak-finding algorithms is often reported to be sensitive to the “kinds” of peak that are present (Wilbanks and Facciotti, 2010). Therefore, developing peak-finding algorithms that perform well in a range of settings remains an open research question.

## 5. Discussion

We have introduced “SMoothing by Adaptive SHrinkage” (SMASH) for smoothing Gaussian and Poisson data using multi-scale methods. The method is built on the empirical Bayes shrinkage method, ASH, whose two key features are: (i) it models the multi-scale wavelet coefficients using a flexible family of unimodal distributions; and (ii) it accounts for varying precision among coefficients. The first feature allows SMASH to flexibly adapt the amount of shrinkage to the data, so data that “look smooth” are more strongly smoothed than data that do not. The second feature allows SMASH to deal effectively with heteroskedastic variances, and consequently the mean gets smoothed more strongly in regions where the variance is greater.

Notably, and unlike many wavelet shrinkage approaches, SMASH is self-tuning, and requires no specification of a “primary resolution level” (e.g., Nason, 2002) or other tuning parameters. This feature is due to the adaptive nature of ASH; when a particular resolution level shows no strong signal in the data, ASH learns this and adapts the amount of shrinkage (smoothing) appropriately. This ability to self-tune is important for two reasons. First, it makes the method easier to use by non-experts, who may find appropriate specification of tuning parameters challenging. Second, it means that the method can be safely applied “in production” to large numbers of data sets in settings such as genomics where it is impractical to hand-select appropriate tuning parameters separately for every data set.

Our results demonstrate that SMASH provides a flexible, fast and accurate approach to smoothing and denoising. We illustrated this flexibility by applying it to two challenging problems: Gaussian heteroskedastic regression and smoothing of Poisson signals. In both cases, our method is competitive with existing approaches.

While SMASH requires more computation than a simple thresholding rule, it is fast enough to deal with large problems. This is partly because fitting the unimodal distribution in ASH is a convex optimization problem that can be solved quickly and stably using existing numerical optimization techniques (Kim et al., 2020; Koenker and Gu, 2017; Stephens, 2017). Using the convex optimization library MOSEK (Friberg, 2017), which is interfaced through the “KWDual” function in the R package REBayes (Koenker and Gu. 2017), fitting the ASH model typically takes about 30 seconds or less for a data set with 100,000 observations. (This timing is based on running R 3.4.3 on a MacBook Pro with a 3.5 GHz Intel i7 multicore CPU and no multithreaded external BLAS/LAPACK libraries.) SMASH requires multiple applications of ASH—it is applied at each resolution level, and requires ${log}_{2}\;(T)$ applications in the Poisson case—yet it remains fast enough to be practical for moderately large problems; for example, smoothing a signal of length 2^15^=32,768 typically takes less than 1 minute for Poisson-distributed data, and less than 2 minutes for Gaussian data. It is likely these runtimes could be further improved by more efficient implementations.

Besides its accuracy for point estimation, SMASH also has the advantage that it naturally provides measures of uncertainty in estimated wavelet coefficients, which in turn provide measures of uncertainty (e.g., credible bands) for estimated mean and variance functions.

Although we have focussed on applications in one dimension, ASH could potentially be applied to multi-scale approaches in higher dimensions, such as image denoising (Nowak, 1999). Alternatives to wavelets, such as curvelets (Candès and Donoho, 2000), may produce better results for image processing applications. Extending our work to those settings could be an interesting direction for future work.

## Figures and Tables

**Table 1:** Accuracy of SMASH and MFVB in two simulation scenarios. In each simulation, accuracy is measured using the mean of squared errors (MSE). The table shows the MSE averaged over the 100 simulations in each of the scenarios. The true mean and standard deviation (s.d.) functions are shown in Figure 5. In Scenario 1, the data are not equally spaced, and the number of data points is not a power of 2; in this setting, SMASH is more accurate in estimating both the mean and s.d. In Scenario 2, the data are equally spaced, and the number of data points is a power of 2; SMASH again outperforms MFVB in both mean and s.d. estimation.

	Scenario 1		Scenario 2	
	MSE (for mean)	MSE (for s.d.)	MSE (for mean)	MSE (for s.d.)
MFVB	0.0330	0.0199	0.0172	0.0085
SMASH	0.0334	0.0187	0.0158	0.0065

## Appendix A. Variance Estimation for Gaussian Denoising

With Z as defined in (16), we apply the wavelet transform W to $Z^{2}$, and obtain the wavelet coefficients $\delta =WZ^{2}$. Note that E(δ)=γ, where $\gamma =W\sigma^{2}$. We treat the likelihood for γ as if it were independent, resulting in

$$L\left(\gamma |\delta \right)=\overset{J}{\underset{j=0}{\prod }}\overset{T-1}{\underset{k=0}{\prod }}p\left(\delta_{jk}|\gamma_{jk}\right).$$

The likelihoods $L\left(\gamma_{jk}|\delta_{jk}\right)$ are not normal, but we approximate the likelihood by a normal density through matching the moments of a normal distribution to the distribution $p\left(\delta_{jk}|\gamma_{jk}\right)$; that is,

$$p\left(\delta_{jk}|\gamma_{jk}\right)\approx N\left(\gamma_{jk},{\hat{s}}^{2}\left(\delta_{jk}\right)\right)$$

so that

$$L\left(\gamma_{jk}|\delta_{jk}\right)\approx \phi \left(\delta_{jk};\gamma_{jk},{\hat{s}}^{2}\left(\delta_{jk}\right)\right),$$

where ϕ is the normal density function, and ${\overset{ˆ}{s}}^{2}\left(\delta_{jk}\right)$ is the variance of the empirical wavelet coefficients. Since these variances are unknown, we estimate them from the data and then proceed to treat them as known. Specifically, since $Z_{t}\sim N\left(0,\sigma_{t}^{2}\right)$, we have that

$$E\left(Z_{t}^{4}\right)\approx 3\sigma_{t}^{4}$$

$$\text{Var}\left(Z_{t}^{2}\right)\approx 2\sigma_{t}^{4},$$

so we simply use $\frac{2}{3}Z_{t}^{4}$ as an unbiased estimator for $Var\;\left(Z_{t}^{2}\right)$. It then follows that ${\overset{ˆ}{s}}^{2}\left(\delta_{jk}\right)$ is given by $\sum_{l=1}^{T}\;\frac{2}{3}Z_{l}^{4}W_{jk,l}^{2}$, and is an unbiased estimate of $Var\;\left(\delta_{jk}\right)$. These will be the inputs to ASH, which then produces shrunk estimates in the form of posterior means for the corresponding parameters. Although this works well in most cases, there are variance functions for which the above procedure tends to overshrink the wavelet coefficients at the finer levels. This is likely because the distribution of the wavelet coefficients is extremely skewed, especially when the true coefficients are small (at coarser levels the distributions are much less skewed since we are dealing a linear combination of a large number of data points). One way around this issue is to employ a procedure that jointly shrinks the coefficients γ and their variance estimates (this is implemented by the jash option in our software). The final estimate of the variance function is obtained from the posterior means via the average basis inverse across all the shifts.

## Appendix B. Poisson Denoising

First, we summarize the data in a recursive manner by defining

$$y_{J,k}\equiv y_{k},$$

for k=1,…,T, with $T=2^{J}$, and

$$y_{jk}=y_{j+1,2k}+y_{j+1,2k+1}$$

for resolutions j=0,…,J-1 and locations $k=0,\ldots ,2^{j}-1$. Hence, we are summing more blocks of observations as we move to coarser levels.

This recursive scheme leads to:

$$y_{jk}=\overset{\left(k+1\right)2^{J-j}}{\underset{l=k2^{J-j}+1}{\sum }}y_{l}$$

for j=0,…,J and $k=0,\ldots ,2^{j}-1$.

Similarly, we define

$$\mu_{J,k}\equiv \mu_{k}$$

for k=1,…,T, and

$$\mu_{jk}=\mu_{j+1,2k}+\mu_{j+1,2k+1}$$

for j=0,…,J-1 and $k=0,\ldots ,2^{j}-1$. And define

$$\alpha_{jk}=\text{log }\mu_{j+1,2k}-\text{log }\mu_{j+1,2k+1}$$

for s=0,…,J-1 and $l=0,\ldots ,2^{j}-1$. The $\alpha_{jk}$ ‘s defined this way are analogous to the (true) Haar wavelet coefficients for Gaussian signals.

Using this recursive representation, the likelihood for α factorizes into a product of likelihoods, where α is the vector of all the $\alpha_{jk}$ ‘s. See Kolaczyk (1999), for example. Specifically,

$$L\left(\alpha |Y\right)=p\left(Y|\alpha \right)\;=p\left(y_{0,0}|\mu_{0,0}\right)\overset{J-1}{\underset{j=0}{\prod }}\overset{2^{j}-1}{\underset{k=0}{\prod }}p\left(y_{j+1,2k}|y_{j,k},\alpha_{j,k}\right)\;=L\left(\mu_{0,0}|y_{0,0}\right)\overset{J-1}{\underset{j=0}{\prod }}\overset{2^{j}-1}{\underset{k=0}{\prod }}L\left(\alpha_{j,k}|y_{j+1,2k},y_{j,k}\right).$$

Note that $y_{00}|\mu_{00}\sim \text{Pois}\;\left(\mu_{00}\right)$. For any given j and $k,y_{jk}$ is a sum of two independent Poisson random variables, and is itself a Poisson random variable. Hence,

$$y_{j+1,2k}|y_{jk},\alpha_{jk}\sim \text{Bin}\left(y_{jk},\frac{1}{1+e^{-\alpha_{jk}}}\right)=\text{Bin}\left(y_{jk},\frac{\mu_{j+1,2k}}{\mu_{jk}}\right).$$

### B.1 Estimates and Standard Errors for $\alpha_{j}$

Each $\alpha_{j}$ is a ratio of the form $log\;\left(\mu_{a:b}/\mu_{c:d}\right)$, whose maximum likelihood estimate (MLE) is $log\;\left(y_{a:b}/y_{c:d}\right)$. The main challenge here is that the MLE is not well behaved when either the numerator $y_{a:b}$ or denominator $y_{c:d}$ is zero. To deal with the case when either is zero, we use Tukey’s modification (Gart and Zweifel, 1967). Specifically, letting S denote $y_{a:b},F$ denote $y_{c:d}$ and N=S+F (effectively treating these as successes and failures in a binomial experiment, conditioned on $y_{a:b}+y_{c:d}$), we use estimator

(21)
$$\hat{\alpha }=\{\begin{array}{ll} \text{log}\left\{\left(S+\frac{1}{2}\right)/\left(F+\frac{1}{2}\right)\right\}-\frac{1}{2} & \text{if }S=0\\ \text{log}\left\{\left(S+\frac{1}{2}\right)/\left(F+\frac{1}{2}\right)\right\}+\frac{1}{2} & \text{if }S=N\\ \text{log}\left(S/F\right) & \text{otherwise} \end{array}$$

(22)
$$se\left(\hat{\alpha }\right)=\sqrt{V^{\text{*}}\left(\hat{\alpha }\right)-\frac{1}{2}V_{3}{\left(\hat{\alpha }\right)}^{2}\left(V_{3}\left(\hat{\alpha }\right)-\frac{4}{N}\right),}$$

where

$$V_{3}\left(\hat{\alpha }\right)=\frac{N+1}{N}\left(\frac{1}{S+1}+\frac{1}{F+1}\right),\;\left(S=0,\ldots ,N\right)$$

$$V^{\text{*}}\left(\hat{\alpha }\right)=V_{3}\left(\hat{\alpha }\right)\left(1-\frac{2}{N}+\frac{V_{3}\left(\hat{\alpha }\right)}{2}\right).$$

The square of the standard error in (22) corresponds to $V^{**}$ from p. 182 of Gart and Zweifel (1967), and is chosen because it is less biased for the true variance of $\overset{ˆ}{\alpha }$ (when N is small) as compared to the asymptotic variance of the MLE (see Gart and Zweifel, 1967). The other two variance estimators from Gart and Zweifel (1967), $V_{1}^{++}$ and $V^{++}$, were also considered in simulations and gave similar results, but $V^{**}$ was chosen for its simpler form.

### B.2 Signal Reconstruction

The first step to reconstructing the signal is to find the posterior means of $p_{jk}:=\frac{\mu_{j+1,2k}}{\mu_{jk}}$ and $q_{jk}:=\frac{\mu_{j+1,2k+1}}{\mu_{jk}}$, for j=0,…,J-1 and $k=0,\ldots ,2^{j}-1$. Specifically, for each j and k, we require

(23)
$$E\left(p_{jk}\right)\equiv E\left(\frac{1}{1+e^{-\alpha_{jk}}}\right)$$

(24)
$$E\left(q_{jk}\right)\equiv E\left(\frac{1}{1+e^{\alpha_{jk}}}\right).$$

Given the posterior means and variances for $\alpha_{jk}$ from ASH, we can approximate (23–24) using the delta method. First, we define

$$f\left(x\right)=\frac{1}{1-e^{-x}},$$

and consider the Taylor expansion of f(x) about f(E(x)),

$$f\left(x\right)\approx f\left(E\left(x\right)\right)+df\left(E\left(x\right)\right)\left(x-E\left(x\right)\right)+\frac{d^{2}f\left(E\left(x\right)\right)}{2}{\left(x-E\left(x\right)\right)}^{2},$$

where

$$df\left(x\right)=\frac{e^{x}}{{\left(1+e^{x}\right)}^{2}}$$

$$d^{2}f\left(x\right)=\frac{e^{x}\left(1-e^{x}\right)}{{\left(1+e^{x}\right)}^{3}}.$$

Therefore,

$$E\left(p_{jk}\right)\approx f\left(E\left(\alpha_{jk}\right)\right)+\frac{d^{2}f\left(E\left(\alpha_{jk}\right)\right)}{2}\text{Var}\left(\alpha_{jk}\right)$$

$$E\left(q_{jk}\right)\approx f\left(-E\left(\alpha_{jk}\right)\right)+\frac{d^{2}f\left(-E\left(\alpha_{jk}\right)\right)}{2}\text{Var}\left(\alpha_{jk}\right),$$

which can be computed by plugging in $E\left(\alpha_{jk}\right)$ and $Var\;\left(\alpha_{jk}\right)$ from ASH.

Finally, we approximate the posterior mean for $\mu_{t}$ by noting that $\mu_{t}$ can be written as a product of the $p_{jk}$ ‘s and $q_{jk}$ ‘s for any t=1,2,…,T. Specifically, let $c_{1},\ldots ,c_{J}$ be the digits of the binary encoding of t-1, and let $d_{m}=\sum_{j=1}^{m}c_{j}2^{m-j}$, for j=1,…,J-1. Then we have that

(25)
$$\mu_{t}=\mu_{00}p_{00}^{1-c_{1}}p_{1,d_{1}}^{1-c_{2}}\cdots p_{J-1,d_{J-1}}^{1-c_{J}}q_{00}^{c_{1}}q_{1,d_{1}}^{c_{2}}\cdots q_{J-1,d_{J-1}}^{c_{J}},$$

where we usually estimate $\mu_{00}$ as $\sum_{l}\;y_{l}$, following Kolaczyk (1999). Further, exploiting the independence of the $p_{jk}$ ‘s and $q_{jk}$ ‘s at different scales, we have that

(26)
$$E\left(\mu_{t}\right)=\mu_{00}E{\left(p_{00}\right)}^{1-c_{1}}E{\left(p_{1,d_{1}}\right)}^{1-c_{2}}\cdots E{\left(p_{J-1,d_{J-1}}\right)}^{1-c_{J}}\times E{\left(q_{00}\right)}^{c_{1}}E{\left(q_{1,d_{1}}\right)}^{c_{2}}\cdots E{\left(q_{J-1,d_{J-1}}\right)}^{c_{J}}.$$

We can also approximate the posterior variance of $\mu_{t}$. (This allows creation of an approximate credible interval under normal approximation.) From (25), we have

(27)
$$E\left(\mu_{t}^{2}\right)=\mu_{00}^{2}E{\left(p_{00}^{2}\right)}^{1-c_{1}}E{\left(p_{1,d_{1}}^{2}\right)}^{1-c_{2}}\cdots E{\left(p_{J-1,d_{J-1}}^{2}\right)}^{1-c_{J}}\times E{\left(q_{00}^{2}\right)}^{c_{1}}E{\left(q_{1,d_{1}}^{2}\right)}^{c_{2}}\cdots E{\left(q_{J-1,d_{J-1}}^{2}\right)}^{c_{J}}.$$

To compute this quantity, we again use the delta method, with $f(x)={\left(\frac{1}{1+e^{-x}}\right)}^{2}$, to obtain:

(28)
$$E\left(p_{jk}^{2}\right)\approx {\left(f\left(E\left(\alpha_{jk}\right)\right)+d^{2}f\left(E\left(\alpha_{jk}\right)\right)\text{Var}\left(\alpha_{jk}\right)/2\right)}^{2}+{\left\{df\left(E\left(\alpha_{jk}\right)\right)\right\}}^{2}\text{Var}\left(\alpha_{jk}\right)$$

(29)
$$E\left(q_{jk}^{2}\right)\approx {\left(f\left(-E\left(\alpha_{jk}\right)\right)+d^{2}f\left(-E\left(\alpha_{jk}\right)\right)\text{Var}\left(\alpha_{jk}\right)/2\right)}^{2}+{\left\{df\left(E\left(-\alpha_{jk}\right)\right)\right\}}^{2}\text{Var}\left(\alpha_{jk}\right).$$

Finally, we combine (26) and (27) to obtain $Var\;\left(\mu_{t}\right)$.

### B.3 Translation Invariance

It is common in multi-scale analysis to perform analyses over all T circulant shifts of the data, because this is known to consistently improve accuracy. (The t-th circulant shift of the signal Y is created from Y by moving the first T-t elements of Y t positions to the right, then inserting the last t elements of Y into the first t locations.)

To implement this in practice, we begin by computing the $\alpha_{j}$ coefficients, and their corresponding standard errors, for all T circulant shifts of the data. This is done efficiently in $O\left({log}_{2}\;T\right)$ operations using ideas from Coifman and Donoho (1995). We took the steps described in Kolaczyk (1999); indeed, our software implementation benefitted from the MATLAB code provided by Kolaczyk (1999) for the TI table construction, which we ported to C++ and interfaced to R using Rcpp (Eddelbuettel and Francois, 2011).

This yields a table of α coefficients, with T coefficients at each of ${log}_{2}\;T$ resolution levels, and a corresponding table of standard errors. As in the Gaussian case, we then apply ASH separately to the T coefficients at each resolution level to obtain a posterior mean and posterior variance for each $\alpha_{j}$. Finally, we use the methods detailed above to compute quantities of interest averaged over all T shifts of the data. For example, our final estimate of the mean signal $\mu_{k}$, for k=1,…,T, is given by $\sum_{t=1}^{T}\;{\overset{ˆ}{\mu }}_{k}^{\left(t\right)}/T$, where ${\overset{ˆ}{\mu }}_{k}^{\left(t\right)}$ denotes the posterior mean of $\mu_{k}$ computed from the t-th circulant shift of the data. Again, borrowing ideas from Coifman and Donoho (1995), this averaging can be done with $O\left({log}_{2}\;T\right)$ operations.

## Appendix C. Implementation of Haar-Fisz method in Poisson simulations

We explored four options for the Gaussian denoising stage of the Haar-Fisz method, all with 50 “external cycle-spins” (Fryzlewicz and Nason, 2004):

1. A hybrid of the greedy tree denoising algorithm (Baraniuk, 1999) and wavelet thresholding using “leave-half-out” cross-validation (Nason, 1995). We used $j_{0}=3$ (the default setting), and the noise level was estimated from the data. These choices correspond to the “H:CV+BT CS” method in (Fryzlewicz and Nason, 2004). In practice, we found that the algorithm did not always converge, in which case we marked the solution as being unavailable.
2. Wavelet thresholding using the universal threshold (Donoho and Johnstone, 1994). We used $j_{0}=3$ (the default setting), and the noise level was estimated from the data. These choices correspond to the “F凶U CS” method in (Fryzlewicz and Nason, 2004).
3. Wavelet thresholding using the universal threshold for the non-decimated wavelet transform. Results were averaged over settings $j_{0}=4,5,6,7$, and the noise level was estimated from the data.
4. Wavelet thresholding using the universal threshold for the non-decimated wavelet transform, in which the noise level was set to 1 rather than estimate it from the data (this is the asymptotic variance under the Fisz transform). Results were averaged over settings $j_{0}=4,5,6,7$.

The settings of each HF method were chosen by us to optimize (average) performance through experimentation on a range of simulations.

## Appendix D. Test functions used to simulate data

Figures 9 and 10 show the mean and variance functions used to simulate the Gaussian data sets. Figure 11 shows the intensity functions used to simulate the Poisson data sets.
